# High-resolution multi-omics enhances prediction and detection of smORF-encoded proteins in the human gut microbiome

**DOI:** 10.1038/s41467-026-72762-5

**Published:** 2026-05-09

**Authors:** Megan E. Davin, Júlia Ortís Sunyer, Luis F. Delgado, Steven L. Tavis, Tuesday Lowndes, Zainab Zafar, Jordan Caussin, Rashi Halder, Oskar Hickl, Cédric C. Laczny, Etienne Hanslian, Daniela A. Koppold, Anika Rajput-Khokhar, Nico Steckhan, Sebastian Schade, Jochen Schneider, Brit Mollenhauer, Andreas Michalsen, Patrick May, Robert L. Hettich, Paul Wilmes

**Affiliations:** 1 Bredesen Center for Interdisciplinary Research, Graduate School of Genome Science and Technology, University of Tennessee, Knoxville, TN USA; 2 Biosciences Division, Oak Ridge National Laboratory, Oak Ridge, TN USA; 3 Luxembourg Centre for Systems Biomedicine, University of Luxembourg, Esch-sur-Alzette, Luxembourg; 4 Institute for Social Medicine, Epidemiology and Health Economics, Charité Universitätsmedizin Berlin, Berlin, Germany; 5 Department of Internal and Integrative Medicine, Immanuel Hospital Berlin-Wannsee Branch, Berlin, Germany; 6 Department of Dermatology, Venereology and Allergology, Charité Universitätsmedizin Berlin, Berlin, Germany; 7 Digital Health-Connected Healthcare, Hasso Plattner Institute, University of Potsdam, Potsdam, Germany; 8 Department of Neurology, University Medical Center Göttingen, Göttingen, Germany; 9 Movement Disorders and Parkinson’s Disease, Paracelsus-Kliniken, Kassel, Germany; 10 Department of Internal Medicine and Psychiatry, Saarland University Hospital and Saarland University Faculty of Medicine, Homburg, Germany; 11 Department of Health, Medicine and Life Sciences, Faculty of Science, Technology and Medicine, University of Luxembourg, Esch-sur-Alzette, Luxembourg; 12 Present Address: Luxembourg Institute of Health, Esch-sur-Alzette, Luxembourg

**Keywords:** Microbial communities, Proteomics, Protein analysis

## Abstract

Small open reading frames (smORFs), which encode proteins under 100 amino acids, represent an underexplored dimension of the human gut microbiome, despite growing evidence of their essential biological roles. Due to small size and poor annotation, smORFs are typically excluded from metagenomic/metaproteomic analyses. Here, we present a high-resolution multi-omic workflow that integrates smORF prediction into metaproteome searches and enables ultra-deep detection of smORF-encoded proteins (SEPs), without experimental size-based enrichment, utilizing state-of-the-art mass spectrometry instrumentation. Applied to human gut microbiomes, this approach resulted in the largest number of detected SEPs to date, allowing identification of over 25,000 SEPs in the metaproteome, alongside the measurements of the larger proteins. Our multi-omics integrative strategy is critical for advancing human metaproteome research. It also provides a generalizable strategy for comprehensive SEP discovery across diverse microbial ecosystems greatly expanding the previously hidden proteomic landscape.

## Abstract (web summary)

Here, the authors develop a new multi-omics workflow that reveals millions of predicted small open reading frames that translate to thousands of identified small proteins by metaproteomics within the human gut microbiome, expanding understanding of microbial biology and enabling more comprehensive metaproteome studies.

## Introduction

The human gut microbiome is a complex ecosystem comprised of bacteria, archaea, microeukaryotes, and viruses that are closely interconnected with the human host. It plays a fundamental role in maintaining human health, influencing processes ranging from nutrient metabolism to immune modulation and neurological function^1^. While extensive research has explored the microbiome’s taxonomic composition and functional output^2,3^, a growing body of evidence points to small open reading frames (smORFs) and their encoded proteins as previously overlooked contributors to microbiome-driven physiology and human health^4–6^.

Human gut microbiome-borne smORFs can currently be predicted using a combination of advanced computational and experimental approaches. Modified versions of the gene caller Prodigal or tools such as smORFinder can be used to predict ORFs as short as five amino acids (aa)^7–9^. In addition, methods such as metatranscriptomics and ribosome profiling can be used to resolve transcription and translation of these smORFs^8,10^. Recent efforts using publicly available metagenomes have predicted tens to hundreds of thousands of smORFs in the human gut microbiome^7,11^. However, these expanded predictions in genomic and transcriptomic data in the literature are rarely followed up with proteomic measurements to validate the smORF-encoded polypeptides or proteins (SEPs) and their context-dependent expression. Currently, there is no universally accepted definition for the length of SEPs, with reported thresholds varying widely across studies^5,7,8,12^. In the present study, we adopt an upper cutoff of 100 aa. This choice aligns with historical genome annotation practices, which commonly apply a 100 aa threshold to exclude the large number of short ORFs that may arise by chance^12,13^. SEPs have historically been excluded from standard annotations and proteomic workflows due to their small size, low sequence conservation, and likely cryptic genomic context^5,14,15^. While smORF prediction is increasing, detection and annotation workflows for proteome search databases to evaluate and confirm smORF predictions remain incomplete. Functional annotation is still limited, and false discovery rates (FDRs) can be high in the absence of rigorous filtering strategies.

Despite these challenges, recent focused studies of SEPs suggest their involvement in cancer, heart disease, and neurological disorders, furthering the need for their inclusion in untargeted analyses^12,16–18^. Furthermore, smORFs and SEPs have been documented as critical players in antimicrobial resistance, ribosomal and translational regulation, and a variety of other essential microbial functions^5,8^. In a large-scale analysis of human metagenomes, over 50% of predicted smORFs were annotated with roles in intercellular signaling, emphasizing their potential impact on host-microbe interactions and, consequently, on human health^7^. The importance of integrating SEP detection within an untargeted metaproteome measurement is further underscored by evidence that smORFs can represent a substantial portion of detected proteins in bacterial isolate cultures. In *Bacillus subtilis*, SEPs account for 12% of all identified proteins^11^. Additionally, a study that used experimental size-filtering found thousands of SEP groups in mouse fecal samples, and over 800 in human tissue samples^19,20^.

Despite mounting evidence of their importance, SEPs typically are not integrated into untargeted proteomic search databases. In general, proteomic studies that detect SEPs use methods to experimentally filter out large proteins^19,21,22^, and while such strategies offer valuable insight into SEP presence, they fail to capture these proteins within the context of the complete proteomic landscape. One reason for the lack of SEP detection is low abundance; SEPs often fall below the detection threshold of traditional mass spectrometry (MS) platforms, due to limitations in sensitivity and dynamic range. New instrumentation, such as the ThermoFisher Orbitrap Astral™, offers greatly improved measurement metrics in speed, throughput, and sensitivity^23^. These advancements increase the likelihood of detecting low-abundance SEPs in complex microbiome samples without requiring pre-enrichment or de novo peptide sequencing, allowing for a more accurate and complete view of their contributions.

Here, we present a workflow which is based on the integration of metagenomics and metatranscriptomics to produce comprehensive metaproteomic search databases with embedded smORF predictions. These databases are then mined with mass spectral data from the newest generation of high-performance MS instrumentation, the Orbitrap Astral, for ultra-deep untargeted metaproteome characterizations, including both large, annotated proteins as well as SEPs within the same measurement. The focus of this study is human fecal microbiome samples from healthy donors to showcase the pipeline’s ability for broad and accurate detection of SEPs within complex untargeted proteomes. This advancement provides a foundation for a robust multi-omics integration strategy for more inclusive and sensitive metaproteomic profiling and opens new avenues for understanding the role of smORFs and their expression products within microbiomes.

## Results

### Maximizing smORF prediction and SEP detection through workflow integration

**Fig. 1:**
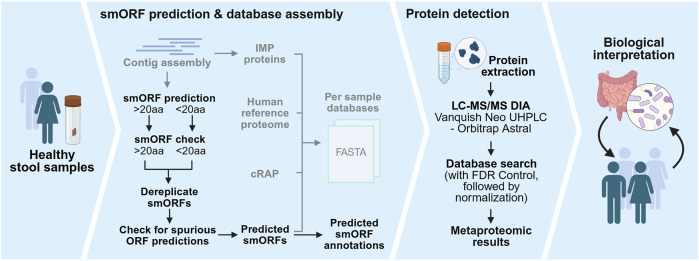
Integrated workflow for small open reading frames (smORFs) prediction, database construction and metaproteomic profiling of healthy human stool samples. Metagenomic and metatranscriptomic reads were processed and assembled using the Integrated Meta-omics Pipeline (IMP), yielding predicted annotated proteins and contigs for downstream smORF prediction. The resulting protein database comprises predicted smORFs, IMP-derived proteins, the human reference proteome, and common contaminants from the common Repository of Adventitious Proteins (cRAP). Created in BioRender. Elliott, M. (2026) https://BioRender.com/uksryyz.

Flash-frozen stool samples from healthy individuals were collected and processed to extract DNA, RNA and proteins, as previously described^23^. The DNA and RNA fractions were sequenced, and the metagenomic (MG) and metatranscriptomic (MT) reads were processed and assembled per-sample using the Integrated Meta-omics Pipeline (IMP)^24^, which yielded predicted annotated proteins as well as the contigs used in the smORF prediction pipeline (Fig. 1; Supplemental Figs. 1 & 2). IMP co-assembles contigs by assembling MG and MT reads in an iterative process, which results in enhanced data usage, improved assembly quality, and increased counts of mappable reads^24^. However, IMP was not designed to specifically detect smORFs. For this reason, incorporating a customized smORF prediction pipeline was crucial to increase the number of SEPs identified in each sample. The smORF prediction pipeline has two branches to optimally predict smORFs smaller or larger than 20 aa (Supplemental Fig. 1). The output of each branch undergoes several checks to minimize false positives. This includes a check for partiality to remove truncated proteins, which ensures that the detected SEPs are not just parts of bigger proteins, and a check against spurious open reading frames (Supplemental Fig. 1). These rigorous checks were used to enhance the quality of the smORF predictions and to reduce the subsequent inclusion of low confidence smORFs. We benchmarked our pipeline against smORFinder using *E. coli* K-12 substrain MG1655 (536 EcoCyc-validated small proteins ≤100 aa, 164 ≤ 50 aa)^5,25^. SmORFinder predicted 17 smORFs ≤50 aa, while our prediction pipeline predicted 450 ≤ 100 aa of which 91 ≤ 50 aa (Supplementary Data 1). Validation with MMseqs2 against EcoCyc, using 100% identity and coverage, found no matches among the smORFs predicted by smORFinder (0/164). When the coverage threshold was lowered to 90%, 16 of 164 smORFs (9.75%) were detected. In contrast, our pipeline identified more smORFs in EcoCyc at 100% identity and coverage: 41 of 164 (25%) for sequences ≤50 amino acids, and 343 of 536 (63.8%) for sequences ≤100 amino acids (Supplementary Data 1)^26^. On the co-assembled contigs, smORFinder predicted 77,027 non-redundant smORFs ≤50 aa, with 628 having metaproteomic detection in our measurements, as detailed below. Our prediction pipeline predicted 524,743 smORFs, with 76,261 (MMseqs2 100% identity ≥90% coverage) also being predicted by smORFinder, and 1912 having been identified in our metaproteomics. The full results and parameters for this section are found in Supplemental Note 1. Overall, our approach substantially improved small protein recovery and validation rates, yielding markedly higher sensitivity against EcoCyc-validated proteins and identifying a greater number of metaproteomics-supported smORFs compared to smORFinder. The predicted smORFs, together with the IMP-derived proteins, the human reference proteome database^27^ and the contaminants database (cRAP)^28^, were used to construct the per-sample metaproteomic search databases (Supplemental Fig. 2). We ensured that the predicted proteins, both from IMP and the smORF prediction pipeline, were exclusively coming from microbial data by removing hits to the human reference genome and cRAP. In addition, a unique SEP ID was devised to identify identical proteins in different samples.

Proteins were extracted from each sample and analyzed using two complementary liquid chromatography tandem mass spectrometry (LC–MS/MS) strategies to compare performance across instrument generations: LC–MS/MS data-dependent acquisition (DDA) on a ThermoFisher Q Exactive Plus mass spectrometer and LC–MS/MS data-independent acquisition (DIA) on a ThermoFisher Orbitrap Astral mass spectrometer. This comparison allowed us to benchmark SEP detection against established workflows while evaluating the enhanced sensitivity of the new Orbitrap Astral platform. The results were then compared and biological interpretations from the Orbitrap Astral data were derived (Fig. 1).

### Prevalence and uniqueness of predicted smORFs

**Fig. 2:**
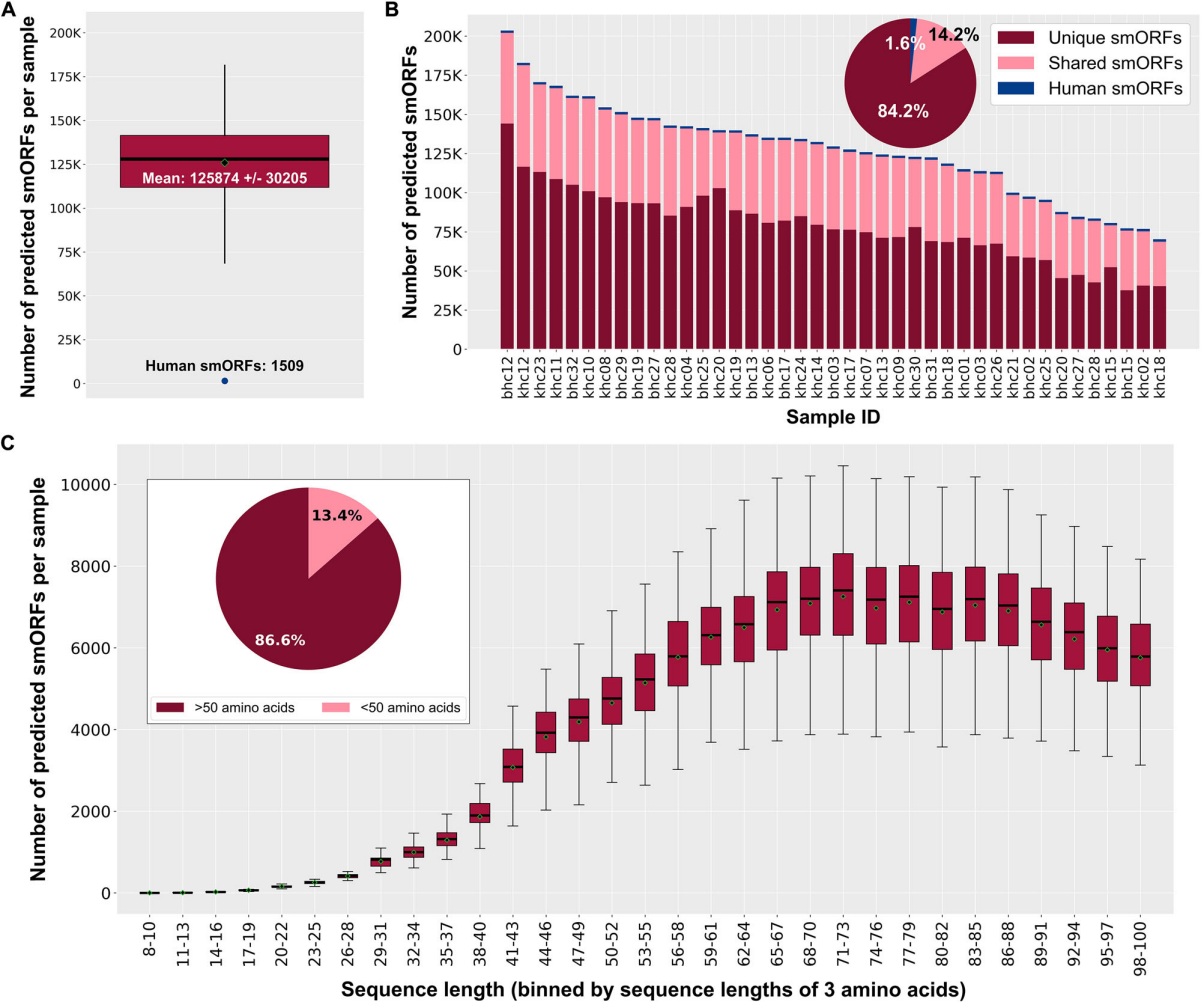
smORF overview. **A** Number of smORFs predicted per sample (*n* = 41). Box plot shows the median (center line), interquartile range (box; 25th–75th percentile), and whiskers extending to the most extreme values within 1.5 × IQR; outliers are not shown. The black diamond indicates the mean. The blue dot indicates the 1,509 human smORFs from the human reference genome database that is included in each sample. **B** Total number of predicted smORFs per sample, including the unique (in dark red) and shared (in pink) predicted smORFs per sample, as well as the human smORFs (in blue). The plot also includes a pie chart showing the distribution of unique, shared and human smORFs across all samples. **C** Sequence length distribution plot of smORFs in each sample using three-amino-acid bins. Each box plot shows the number of predicted smORFs of a determined length per sample (*n* = 41). Box plots show the median (center line), interquartile range (box; 25–75th percentile), and whiskers extending to values within 1.5 × IQR; outliers are not shown. The black diamond indicates the mean. The pie chart shows the proportion of smORFs that have a length bigger or smaller than 50 aa.

Our per sample metaproteomics database contains a total of 5,160,834 predicted smORFs in the sample set, with an average of 125,874 smORFs predicted per sample (Fig. 2A). The minimum amount of smORFs predicted per sample was 68,721, while the maximum was 201,998 smORFs (Fig. 2B). Most of the predicted smORFs were unique to each sample (Fig. 2B). There was a total of 3,218,263 unique smORFs and 542,333 smORFs present in at least 2 samples, with only 49 core smORFs present in all the samples, yielding a total of 3,760,596 non-redundant smORFs (Supplementary Data 2). The predicted smORFs had lengths ranging from 8 to 100 aa, with only 5457 sequences below 20 aa (Fig. 2C**;** Supplemental Fig. 3). 87% of the smORFs were larger than 50 aa, while only 13% of the predicted smORFs had a length of 50 aa or less (Fig. 2C). The five most common smORF sequence lengths were 72, 69, 73, 85, and 79 aa, whereas the five least frequent were 8 to 12 aa (Supplemental Fig. 3**;** Supplementary Data 3). For the 49 core smORFs, the sequence length distribution ranged from 27 to 92 aa, with a median length of 63 aa (Supplemental Fig. 4A).

To evaluate the contribution of MG and MT data to smORF predictions, we mapped MG and MT reads to the predicted smORFs coding sequences and classified smORFs based on presence/absence of mapped reads. Across all samples, 64% of smORFs were supported exclusively by MG reads, 11% exclusively by MT reads, and 25% by both, highlighting the added value of integrating MT data in the contig assembly for comprehensive smORF predictions.

The human reference genome database from NCBI was included in the per-sample metaproteomic databases to detect human proteins. It contained 1509 smORFs, which were therefore included in each per-sample database. The sequence length distribution of the human smORFs ranged from 12 to 100 aa, with the median length being 82 aa (Supplemental Fig. 5A). To confirm minimal human-microbial smORF homology, that could result in the misassignment of spectra to the wrong peptides, we clustered a comprehensive human smORF catalog, (reference proteome smORFs^27^ + 7554 from Martinez et al.^29^ + 150,802 from OpenProt^30^; clustered via MMseqs2^26^ at multiple identity/coverage thresholds) with microbial smORFs. There were no mixed clusters at stringent thresholds (100% identity / 100% coverage and 95% identity / 95% coverage) and only 4 at relaxed thresholds (90% identity / 80% coverage; Supplementary Data 4), mapping to conserved families (Ras/RAB2B, histone H3 variants, ubiquitin-like). The latter thresholds were more relaxed than the parameters typically used in microbiome smORF studies, which commonly use identities and coverages between 90 and 95%^7,8^. With minimal overlap confirmed, subsequent analyses focused on microbial smORFs/SEPs. Full catalog details, results, and parameters in Supplemental Note 2.

We clustered the metaproteomics database at 95% identity and 95% coverage, as used previously in other studies^5^. The clustering yielded 3,060,759 representative smORFs, representing an 18.6% reduction from the original database (Supplementary Data 5). Of these clusters, 86.5% were singletons, while 13.5% formed multi-member clusters with a mean size of 1.23 (Supplementary Data 5). Clustered smORFs showed increased sample prevalence, with 22.2% of the smORFs being found in two samples or more (Supplementary Data 5). This approach reduces complexity while preserving biological diversity, thereby allowing the grouping of functionally similar protein variants together for metaproteomic biological interpretation.

### Taxonomic and functional assignment of predicted smORFs

**Fig. 3:**
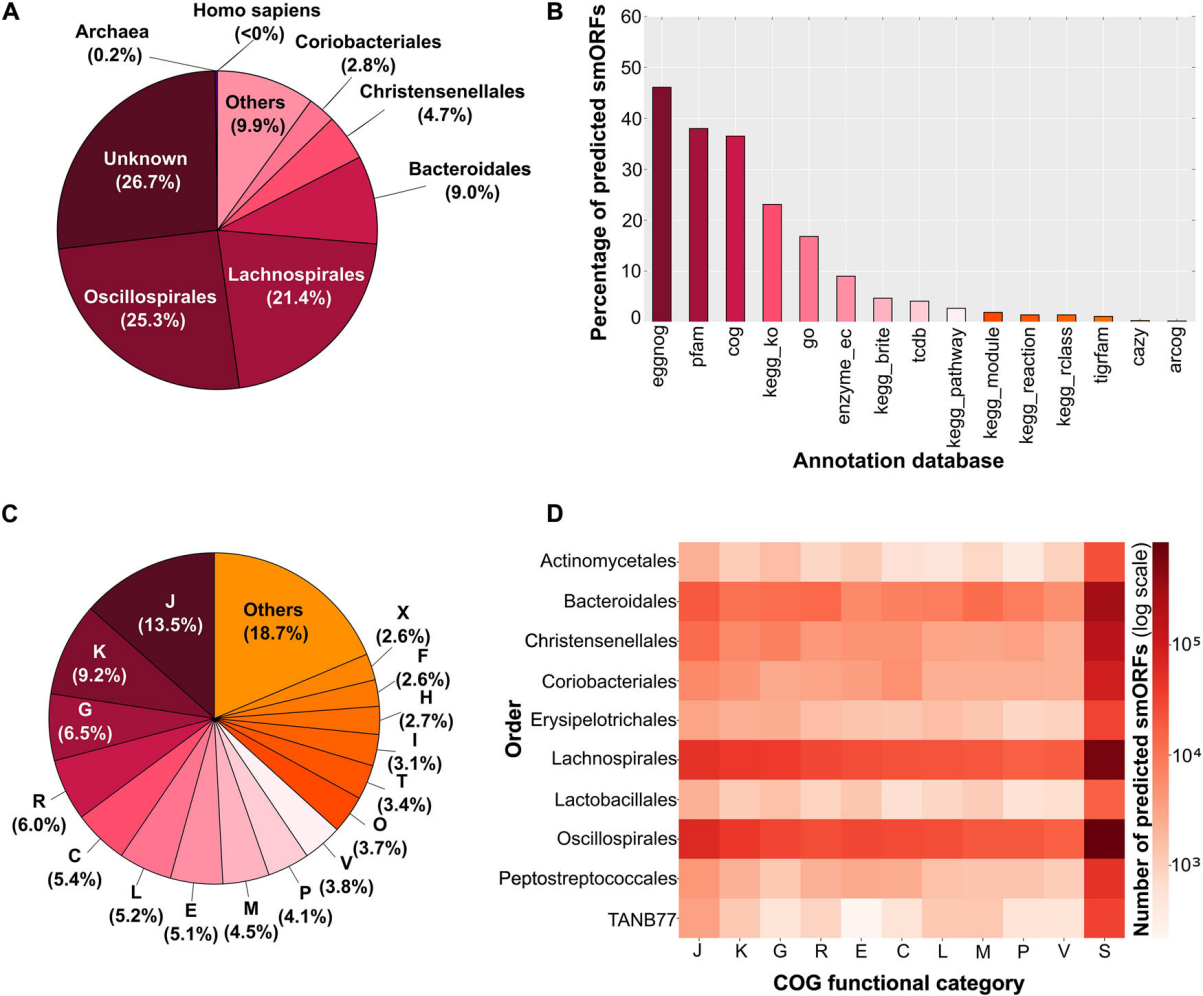
Taxonomic and functional annotations of predicted smORFs. **A** Taxonomy of the predicted smORFs at the order level. All archaeal taxa are grouped together under “Archaea”, and orders representing less than 2% of smORFs are grouped together under “Others”. **B** Percentage of predicted smORFs annotated for each of the databases considered by Mantis for predicted smORFs. **C** COG functional categories for the predicted smORFs. The COG annotations that represent less than 2% of the smORFs are grouped together under “Others” (in light orange). The functions shown, from most to least frequent, are “J: translation, ribosomal structure and biogenesis”, “K: transcription”, “G: carbohydrate transport and metabolism”, “R: general function prediction only”, “C: energy production and conversion”, “L: replication, recombination and repair”, “E: amino acid transport and metabolism”, “M: cell wall, membrane, envelope biogenesis”, “P: inorganic ion transport and metabolism”, “V: defense mechanisms”, “O: posttranslational modification, protein turnover, chaperones”, “T: signal transduction mechanisms”, “I: lipid transport and metabolism”, “H: coenzyme transport and metabolism”, “F: nucleotide transport and metabolism”, “X: mobilome (prophages and transposons)”. **D** Number of smORFs according to the microbial orders (in log scale) with most smORFs with their corresponding functional categories. Only the top ten orders and functional categories plus function “S: unknown function” are shown.

In all predicted smORFs in our per-sample metaproteomics database, two domains, 101 phyla, 299 classes, 767 orders, 1603 families, 5713 genera, and 22,824 species were represented. The taxonomic classification revealed that 79.5% of the sequences could be assigned to known domains. Out of the 4,100,030 classified smORFs, only 10,340 (0.2%) were archaeal, with the rest being bacterial. For this reason, the following taxonomic analysis is focused on bacterial smORFs. Among the bacterial sequences, the phylum Bacillota was the most prevalent, accounting for approximately 3,191,024 (61.8%) smORFs. The most abundant class was Clostridia, with 2,968,916 (57.5%) smORFs. At the order level, Oscillospirales and Lachnospirales, both Clostridia, were the most prevalent with 1,307,040 (25.3%) and 1,105,223 (21.4%) smORFs, respectively (Fig. 3A). At the genus level, *Blautia*, *Faecalibacterium* and *Bacteroides* were the most prevalent genera (Supplementary Data 6).

At the functional level, most of the predicted smORFs remained unannotated; only 2,519,034 (48.8%) smORFs had at least one functional annotation from any functional database. Within each of the broader databases, such as eggNOG, Pfam, or COG, up to 2,380,134 (46.1%) smORFs were annotated. Other more specific databases, such as ArCOG, CAZy or TIGRFAM, had less annotation coverage, with only 11,118 (0.2%) smORFs having an ArCOG annotation (Fig. 3B). The low functional annotation coverage is a common problem when working with smORFs^8^. The smORFs with a COG annotation had diverse functions, with all 26 single-letter categories and 116 composite categories describing the various functionalities of the smORFs (Fig. 3C). The most common functions of the annotated smORFs were “J: translation, ribosomal structure and biogenesis”, “K: transcription” and “G: carbohydrate transport and metabolism” (Fig. 3C). This was also reflected in the orders Lachnospirales and Oscillospirales (Fig. 3D).

All 49 core smORFs had both taxonomic and functional annotations (Supplementary Data 7 & 8). Taxonomically, all the core smORFs were bacterial, and represented two phyla, three classes, four orders, six families, 18 genera and 26 species. At the order level, all core smORFs were assigned taxonomically with most of the smORFs being Bacteroidales, with Lachnospirales, Oscillospirales and Erysipelotrichales also being represented (Supplemental Fig. 4B). All the smORFs were assigned to at least one of the functional databases used by Mantis (Supplemental Fig. 4C). Most of the smORFs had COG functions related to “J: translation, ribosomal structure and biogenesis”, “K: transcription”, “C: energy production and conversion” and “I: lipid transport and metabolism”, with some of them having an “S: unknown function” (Supplemental Fig. 4D). The predominant function in Bacteriodales, Lachnospirales and Oscillospirales was “J: translation, ribosomal structure and biogenesis”, with most core smORFs having an “S: unknown function”, especially in Erysipelotrichales (Supplemental Fig. 4E).

Most of the human smORFs were assigned to at least one of the functional databases used by Mantis (Supplemental Fig. 5B). Due to COG being specific to bacterial and archaeal functions^31^, Pfam was used instead. 1072 (71%) human smORFs could be assigned a Pfam domain, with 515 domains being represented. The most common domains were PF00096 (zinc finger, C2H2), PF00048 (small cytokines, interleukin-8 like), PF13841 (beta defensin) and PF01423 (LSM domain), which were assigned to 51, 32, 28 and 18 human smORFs, respectively. The rest of the Pfam domains were assigned to less than 1% of the smORFs (Supplemental Fig. 5C).

To assess the coverage of our predicted smORFs in a global reference resource, we aligned all smORFs in our metaproteomics database against the Global Microbial smORF Catalog (GMSC) using GMSC-mapper^8,32^. Overall, 3,382,553 smORFs (89.95%) had homologs in GMSC, indicating substantial overlap with the current global catalog; of these, 706,802 (20.90%) were classified as high quality, reflecting homology to a reference smORF that passed stringent confidence filters in the GMSC. Habitat, taxonomic, and domain annotations were transferred from matched GMSC entries: most smORFs were associated with multiple habitats (88.79%), predominantly the human gut (98.66%), whereas 97.08% received taxonomic annotations, and 12.53% contained conserved domains. Notably, metaproteomics data provided experimental support for 1,585 predicted smORFs lacking homologs in GMSC.

### Detection of SEPs within the metaproteome

Untargeted proteomics measurements of all samples were performed using the high-performance Orbitrap Astral mass spectrometry instrumentation. To capture biological variability, metaproteomes were searched against 100% identity and 100% coverage sample-specific databases. Then, as detailed above, peptides were clustered at 95% sequence and coverage similarity for protein-level analysis. This clustering threshold groups highly homologous SEPs allowing for accurate protein counts across samples. This resulted in an average of 84,581 peptides or 27,025 ( ± 2,795) proteins per sample, totaling 332,164 proteins (Supplemental Fig. 6A). These proteins range in lengths, from 21 aa to 29,339 aa, with a median length of 309 aa (Supplementary Data 9). To examine the SEP profiles specifically, all peptides that mapped to both a SEP and a protein over 100 aa were not further considered; this ensured that the peptides analyzed are indeed unique to SEPs and resulted in an average of 2,599 unique peptides mapping to SEPs (2.8% of the average total peptide count) per sample.

**Fig. 4:**
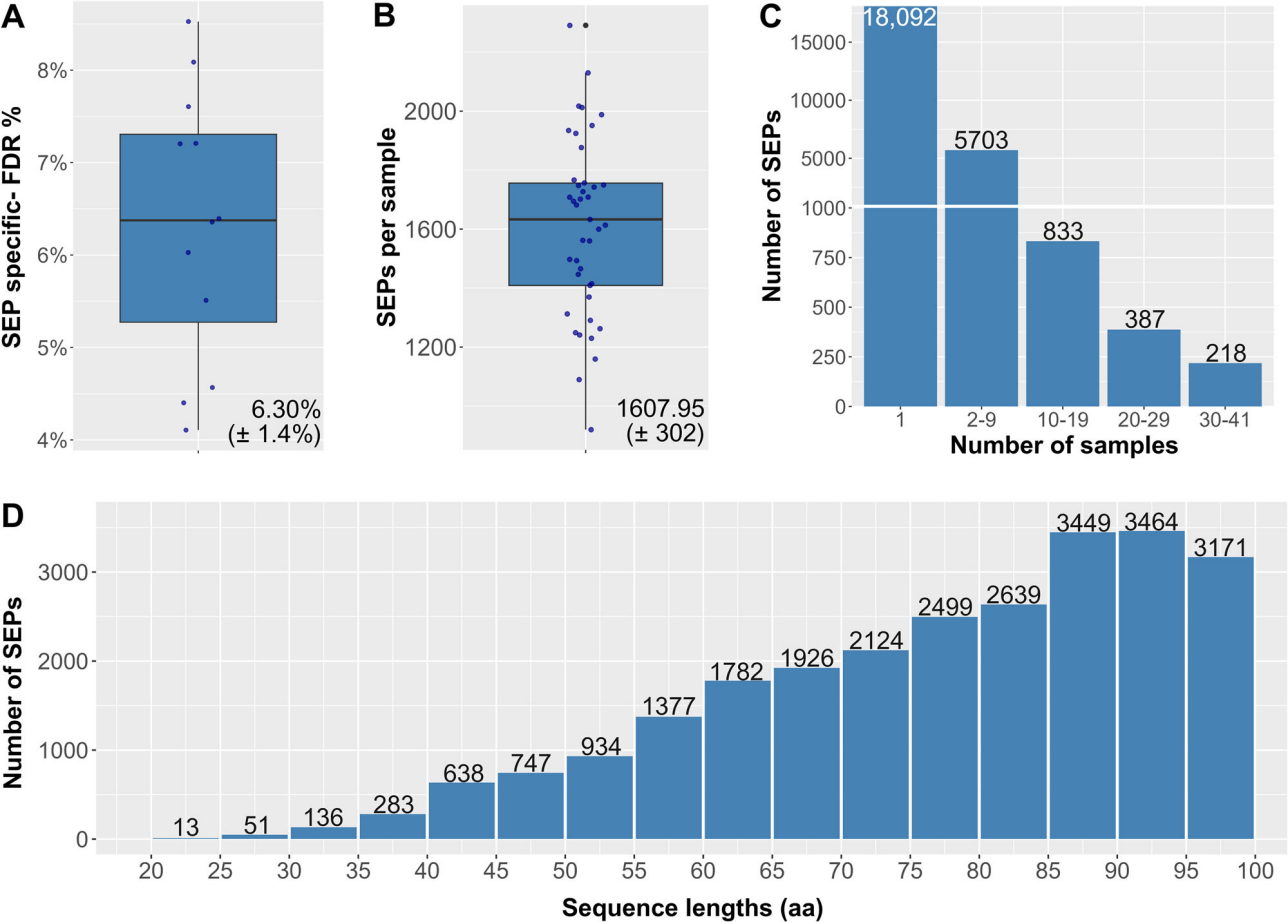
Detection of SEPs using an orbitrap astral instrument. **A** Distribution of the upper-bound SEP-specific (peptide level) False Discovery Rates (FDRs) calculated using a SEP-specific entrapment database (*n* = 12) [see Methods]. Box plot shows individual data points over the median (center line), interquartile range (box; 25th–75th percentile), and whiskers extending to 1.5 × Inner quartile range (IQR). Mean values ± STD are reported in the bottom right corner. **B** Distribution of the total SEPs detected per sample (*n* = 41). Box plots show individual data points over the median (center line), interquartile range (box; 25th–75th percentile), and whiskers extending to values within 1.5 × IQR. Mean values ± STD are reported in the bottom right corner. **C** SEP presence across samples in 1, 2-9, 10-19, 20-29, or 30-41 samples. A plot break is denoted by a white gap at 800 proteins. **D** Number of detected SEPs across the entire sequence length distribution (bin width=5) for all herein detected SEPs. Respective SEP counts are listed above each bar in C/D.

The detected SEPs in these samples maintained a SEP-specific (peptide level) FDR (achieved with a shuffled entrapment database approach) across samples at a mean FDR of 6.3% ( ± 1.4%), validating that the large number of SEPs detected reflect confident identifications (Fig. 4A). An average of just over 1,600 SEPs were detected per sample (representing 7.6% of the total proteome protein count), with a cumulative total of 25,233 unique SEPs across the sample set (Fig. 4B, C). These numbers exceeded previous SEP detections reported in the human gut microbiome and elsewhere, either from studies that employed size-based enrichment of small proteins or those that measured the entire proteome^19,20^. Additionally, SEPs accounted for up to 6.3% of the untargeted proteome abundances in individual samples, with a mean contribution of 3.6% ( ± 0.9% standard deviation). The majority of these SEPs clustered within the 85–100 aa range, representing 40% of all detected SEPs. In contrast, only 13 SEPs below 25 aa in length were detected, with the smallest detected SEPs measuring at just 21 aa, even though our prediction pipeline included smORFs as short as 7 aa (Figs. 2B and 4D).

Comparative analyses against DDA acquisition using the Q Exactive Plus instrumentation further highlighted the superior performance of the Orbitrap Astral instrument. The same samples analyzed with the Astral Orbitrap were run with the Q Exactive Plus. The results of these runs yielded a slightly higher SEP-specific (peptide level) FDR of 7.4% ( ± 1.9%), but just one-third of the number of SEPs, averaging 556 per sample and totaling 8,332 unique SEPs (Supplemental Fig. 7). Furthermore, the proportion of conserved SEPs were lower with Q Exactive Plus measurements: only 0.6% of SEPs were found in 30 or more samples, compared to 0.9% using the Orbitrap Astral (Supplemental Fig. 8). Despite these differences in detected SEPs, the overall sequence length distribution and the smallest detectable peptide length remained consistent between the two platforms.

**Fig. 5:**
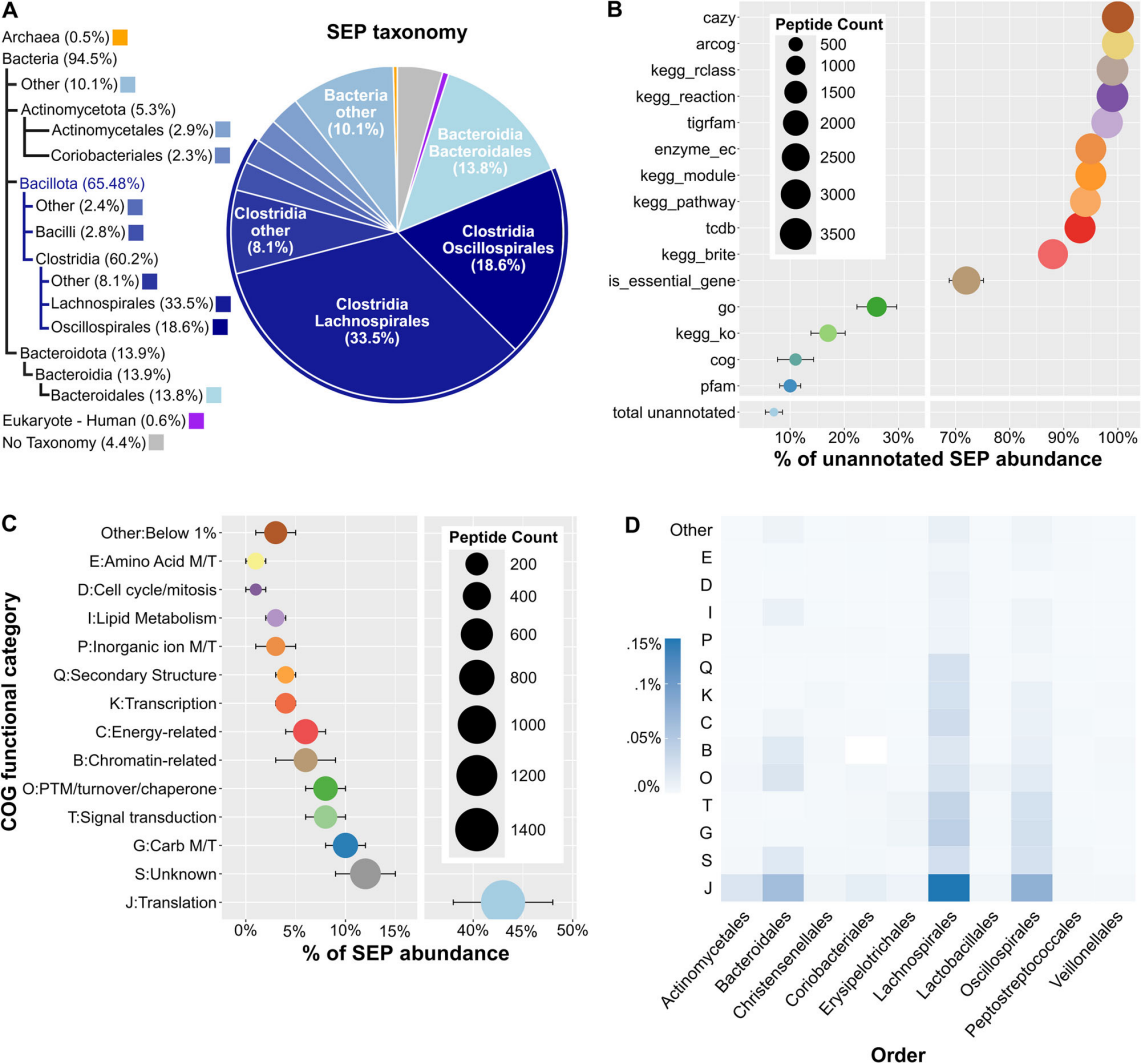
Taxonomic and functional assignments of SEPs. **A** Average taxonomic composition of SEPs by abundance are shown down to order level (mean; *n* = 41). The blue outline around the chart corresponds to the contribution of the phylum Bacillota the SEP proteome. The “other” categories in the legend accounts for SEP abundance that either did not have a more specific classification or did not meet the abundance threshold listed: If the taxonomic order was >2% than it was shown, if not the abundance was rolled up into the higher taxonomic class until it contributed to a taxonomy >2%. Only archaeal, bacterial, and human (*Homo sapiens*) taxonomy was annotated. Human proteins are represented in the “Eukaryote - Human” category denoted in the legend. **B** Average fraction of unannotated SEP abundance per sample for 15 functional annotation databases. Row “total unannotated” (separated by a horizontal graph break) represents protein abundance that does not fit within any of the 15 annotation types represented. A vertical graph break is present between 35% and 65%. Eggnog Annotations are not included as they represent orthologues not functional categories. Dot size is dependent on average number of contributing peptides (mean), and sits at the mean percent of abundance (*n* = 41; error bars ± STD). **C** SEP abundance by COG functional categories. All COG categories are represented by their letter followed by a brief description. A graph break is present after 15% to 35%. Dot size is dependent on average number of contributing peptides (mean), and sits at the mean percent of abundance (*n* = 41; error bars ± STD). **D** A heatmap of the 10 taxonomic orders that were the top contributors to smORF abundance, and their percentage makeup of the SEP profile for different COG categories. In panels C/D, Peptides without a COG category annotation were summed with those mapping to “S: Unknown” or “S”.

The distribution of SEPs across samples revealed interesting patterns of conservation. Most detected SEPs were sample-specific, with 18,092 total SEPs mapping uniquely to just one sample. Within all samples, only 605 SEPs (2.3% of all detected SEPs) appeared in 20 or more of the samples (Fig. 4C). This number of conserved SEPs is comparable to what is seen in the global data, whereby only 3.2% of proteins are detected in 20 or more samples (Supplemental Fig. 6B). Among these, the smallest is a 37 aa long ribosomal protein detected in 20 samples identified as Actinomyces bacterium, *Parolsenella catena* protein. When looking at conservation within Clostridia Lachnospirales, the taxonomic order with the highest SEP abundance (Fig. 5A), SEPs identified in 20 or more samples represent 3.2% of the total identified Lachnospirales SEPs, similar to the percentage of total Lachnospirales proteins (including proteins above 100 aa) identified in 20 or more samples, which was 5.0%. This shows the slightly more conserved nature of Lachnospirales proteins compared to the total SEP and global proteome profiles (Supplemental Fig. 9). When examining the peptides that mapped to the sequences associated with core predicted smORFs, with 100% sequence similarity, 17 of the 49 core smORFs showed detectable protein expression in at least one sample, 7 of these appearing in 20 or more samples.

SEPs that are more conserved tend to contribute more to the overall SEP abundance. The SEPs present in ≥20 samples were identified and analyzed on a per sample basis, on average these “conserved SEPs” make up only 25% ( ± 4.1) of the number of SEPs detected but account for 46% ( ± 9.4%) of total SEP abundance. This disparity grows when looking at the SEPs present in ≥30 samples, per sample they contribute to 11.6% ( ± 2.0%) of total SEPs detected and account for 29% ( ± 6.6%) of the total SEP abundance. Looking at these conserved SEPs within the context of the global proteome, noting this is only possible since our SEP detection was done simultaneously with detection of the global proteome, the untargeted proteome was filtered to include only proteins present in at least 30 samples and within the 50% most abundant fraction of the proteome. This resulted in a set of 1754 proteins, of which ~5.1% (91 proteins) were SEPs.

### Taxonomic and functional analysis of detected SEPs

Across all detected SEPs, a wide range of taxonomic diversity was observed, including 38 phyla, 73 classes, 143 orders, 269 families, 942 genera, and 2793 species (Supplementary Data 10). Within this taxonomic diversity, 87% of SEP abundance could be assigned at least to the phylum level and an average of 4.4% of SEP abundance was not assigned any taxonomic classification (Fig. 5A).

When examining the broader taxonomic profile of these SEPs, we found that only about 1.1% of the protein abundance belonged to archaea or human. The vast majority, 94.5%, originated from bacteria, with Clostridia representing a dominant fraction; 60.2% of the total SEP abundance was assigned to this class. The dominance of Clostridia in the SEP profile parallels the broader taxonomic patterns of global untargeted proteome measurements in this study as well as previous studies^33,34^. Notably, consistent with the predicted smORF-profile, the genus *Blautia*, belonging to the Lachnospirales order and the Lachnospiraceae family accounts for 5.9% of SEP abundance. Overall, the taxonomic composition of the small protein identifications is consistent with that observed in the global metaproteome (including proteins >100 aa), validating that we are not observing spurious small protein hits but rather biologically meaningful patterns (Supplementary Data 11).

When examining SEP abundance, per sample, within any single functional annotation database, an average of 31% ( ± 37%) were annotated (Fig. 5B; Supplementary Data 10). Among the 15 annotation databases considered in this study, four stood out for their relatively high coverage: Pfam, COG, KEGG orthologs, and Gene Ontology. Within these four annotation systems, an average of 16% of SEPS were classified as unannotated per sample (Fig. 5B). While this potentially makes them the most informative for understanding the functional landscape of these SEPs, it is important to note that these tools were not specifically designed for short proteins, and as such, the assigned annotations should be interpreted with caution. It should also be noted that among these five annotation databases, domains of unknown function (DUFs), and less descriptive high-level annotations (such as general metabolism annotations) were among those considered as annotated proteins.

Based on COG annotations, on average per sample, nearly half ( ~ 43%) of the peptides mapping to SEPs detected were related to translation (Fig. 5C), including several well-characterized small ribosomal proteins. These functional categories were expected and provide an important ground-truth, confirming that the workflow captures known as well as novel SEPs. Beyond this dominant category, SEPs were also enriched in carbohydrate metabolism and transport, followed by signal transduction functions, suggesting roles in how microbes process nutrients and respond to environmental cues.

When examining Pfam annotations, the database with the highest annotation rate, categories that were consistently found across samples and highly abundant included: ribosomal subunits, chaperonin subunits, phosphotransferase system proteins, bacterial microcompartments, and FeoA domain proteins (Supplemental Fig. 10). Pfams connected to other important functional activities that were detected at lower abundance but were detected across all samples included categories linked to fatty acid metabolism, redox balance, sporulation, and growth inhibition mechanisms such as antitoxin systems.

The functional profile of the SEP’s differed from that of the global proteome (Supplementary Data 11). Although the translational COG category was the most abundant in the global dataset, similar to the SEP profile, it accounted for only about 20% of total protein abundance on average, representing less than half the proportion observed in the SEP profile. Additionally, the top Pfam categories, in the global dataset, were annotated as elongation factor Tu and glyceraldehyde 3-phosphate dehydrogenases, which were not present at above 1% in the SEP profile. This highlights the importance of including SEPs into the global analysis for a more complete look at the functional profile.

Among the top ten taxonomic orders that contributed to SEP abundance profile, four belonged to the Clostridia class (Fig. 5D), and their individual contributions to functional COG categories closely reflected the untargeted SEP profile (Fig. 5C), with Lachnospirales and Oscillospirales, standing out as the two orders with the highest average SEP abundance (Fig. 5D). Across these top ten orders, all seven core SEPs detected in every sample were represented; three originated from Bacteroidales (Bacteroidaceae family), three from Lachnospirales (Lachnospiraceae family), and one was only resolved to the Bacillota phylum. Functionally, these core proteins included two ribosomal subunit proteins, one cold shock protein, one DNA-binding protein, one acyl-carrier protein, and one bacterial microcompartment (BMC) protein, while one protein remained unannotated. Notably, the cold shock and BMC proteins were highly prevalent, both appearing in over 90% of samples, respectively.

## Discussion

The definition and length threshold for smORFs in prokaryotes remains a subject of ongoing debate within the scientific community. While tools such as smORFinder^5,7^ adopt a maximum length cutoff of 50 aa to define a smORF, our findings suggest that this threshold is too restrictive. More specifically, in the present work, 86.6% of smORFs fell within the size range between 50 to 100 aa, even with filtration against partial smORFs in place. This observation aligns with other recent studies, most notably the microbial smORF catalog (GMSC)^8^, which also uses a 100 aa cutoff to capture a broader spectrum of SEPs. Taken together, our findings support the hypothesis that extending the upper boundary for smORF prediction to 100 aa provides a more inclusive and biologically relevant framework, increasing the likelihood of detecting novel functional elements that may otherwise remain overlooked.

Here, we also established a lower length threshold of five aa for the prediction of smORFs. This decision was based on biological precedent and methodological considerations. The smallest known functional protein to date, the TAL protein from *Drosophila*, is only eleven aa in length^35^. In addition, the search algorithm platform used for the metaproteomics analysis, Proteome Discoverer, sets a threshold of seven aa as the smallest peptide to be detected, while other tools, such as smORFinder and the GMSC, use thresholds of five and ten aa, respectively^8^. Thus, by adopting this lower bound, we aimed to maximize the smORF prediction and ensure that even the shortest potentially functional peptides are not excluded from our analysis.

In addition to setting a permissive lower threshold, we also divided our prediction pipeline into two distinct branches: one targeting smORFs shorter than 20 aa, and another for those equal to or longer than 20 aa. Two branches were implemented due to the gene caller constraining how much two predicted genes are allowed to overlap. This constraint is tied to the minimum gene length setting, so a single set of parameters cannot simultaneously maximize recovery of very short smORFs and longer smORFs without biasing one group. Splitting the workflow allowed us to tune parameters separately for ultra-short and longer smORFs while respecting the gene caller’s overlap rules^14,36^. Extensive gene overlaps create evolutionary constraints by imposing dual coding requirements on the same DNA sequence, where mutations must satisfy the functional needs of both overlapping genes simultaneously^37,38^. If a single pipeline was used with a small maximum overlap setting, longer smORFs could be inadvertently missed due to overlap conflicts. This would be particularly problematic for constructing a comprehensive database for smORF detection at the metaproteomic level, where missing longer smORFs can lead to significant underrepresentation of biologically relevant peptides. Therefore, by implementing two separate prediction strategies, we were able to optimize detection across the full spectrum of smORF lengths while adhering to the technical constraints of the prediction software. This approach ensures that both ultra-short and moderately sized smORFs are comprehensively included in the smORF database. To counteract the high numbers of false positives that are expected by removing Prodigal’s lower thresholds, several false positive checks were implemented: a check for partiality, presence of RBS motifs and for matches against databases containing spurious ORF annotations. RBS motifs were used as a validating metric because it has been previously reported that 73.8% of smORFs contain an RBS site^39^. While this requirement could miss some potential smORFs, it was a necessary quality filter to minimize false positives. This led to the prediction of more than 3.7 million non-redundant smORFs in our human gut microbiome samples, which is substantial, especially in comparison to the 2 million smORFs predicted by Sberro et al.^7^ in human mouth, gut, vagina and skin microbiomes. Mapping our non-redundant predicted smORFs to the GMSC revealed that 89.95% have detectable homologs, underscoring the strong concordance between our predictions and existing global smORF resources. Importantly, the remaining smORFs absent from GMSC are unlikely to represent false positives, as they passed multiple stringent filtering steps, including Antifam, Aragorn, and Infernal screening, as well as checks for partiality and the presence of ribosome binding site motifs. Moreover, a subset of these GMSC-absent smORFs (1585) was independently validated by our metaproteomics data, providing experimental support for the existence of potentially novel smORFs not yet captured in current reference catalogs.

Although developed for gut metagenomic assemblies rather than isolate genomes, our pipeline outperformed smORFinder on *E. coli* K-12 MG1655 under both stringent and relaxed criteria, with consistent boundary prediction and expected short-gene limited recovery^40^. On the co-assembled contigs, most smORFinder predictions overlapped with ours, though only a subset showed metaproteomic support. Benchmarking details and full discussion are available in Supplemental Discussion 1.

Expanding the human smORF catalog for metaproteomics to 157,901 dereplicated sequences yielded few smORFs shared between microorganisms and humans. Using loose matching criteria homologous sequences were restricted to core families like histones, ubiquitin-like systems, and Ras GTPases^41–43^. This rarity of cross-kingdom homology indicates lineage-specific evolution and negligible misassignment risk from using only 1509 reference human smORFs, as shared sequence space is limited. Full details can be found in Supplemental Discussion 2.

Considering peptides unique to SEPs, we found that the average SEP-specific false discovery rate (FDR; at the peptide level) hovered just above the commonly accepted threshold of 5%, supporting the robustness of our detection workflow^44^. Using common protein isolation and preparation methods combined with the Orbitrap Astral this paper, to our knowledge, presents the largest number of SEPs detected, outperforming the known untargeted and targeted microbiome SEP detection currently available. In light of this, the identification of approximately 1600 SEPs per sample and a total of 25,233 SEPs represents a substantial and biologically meaningful part of the untargeted proteomic dataset.

Taxonomic profiles derived from SEP data closely resemble those reported in previous untargeted gut proteomic studies, with both consistent and dominant representation of Clostridia^45^. The concordance between our findings and established microbial profiles supports the validity of the detected smORFs by reducing the likelihood that the observed SEP detection is attributable to random or spurious matches. The detected taxonomic species account for only a fraction of what is seen in the metagenomic data. Metagenomes are typically much deeper than the measure of metaproteomes because metagenomics provides total genomic inventories while metaproteomes measure context-dependent protein expression, which is often greatly reduced relative to total genome potential. In addition, metaproteomics is unable to utilize amplification methods, limiting the dynamic range of the measurements, and furthermore, high-resolution taxonomic assignments are challenging in metaproteomics due to genomes within more-specific taxonomic ranks (i.e., family, or species) having high similarity.

Functionally, SEPs are enriched in key processes central to microbial physiology and host-microbe interactions; however, existing annotation types are derived from and thus developed for longer proteins, highlighting the need to adapt the types to better represent SEPs and their functions. Notably, many SEPs were annotated as established ribosomal proteins, which play important roles in translation. Previous research has also detected small ribosomal proteins within outer membrane vesicles, implicating them in interspecies communication and microbial competition^23^. Additional functional SEP annotations point to roles in bacterial colonization and survival within the gut. For example, SEPs annotated as components of bacterial microcompartments may support bacterial colonization and metabolic specialization^46,47^. Others involved in carbohydrate metabolism, including the phosphotransferase system, may contribute to nutrient processing and have been implicated in host metabolic conditions such as insulin resistance and acute pancreatitis^48–50^. Anti-toxin SEPs, such as the MazE antitoxin were present in every sample; as part of a toxin–antitoxin module, it plays a key role in regulating bacterial growth under stress^51,52^. Redox-related SEPs, which influence oxidative stress responses, also have potential relevance to microbial dysbiosis^53^. Together, these findings highlight that SEPs are not merely present but are functionally intertwined with both core microbial processes and key axes of microbiome–host interactions, underscoring their importance in shaping gut ecosystem dynamics.

Despite their small size and usual lack of inclusion in protein search databases, SEPs represent a portion of the dark metaproteome but undoubtably play critical roles in microbial function, competition, and potentially host interactions. Here, we present a customized pipeline for high-quality, comprehensive smORF prediction that uses co-assembled MG/MT contigs, a broader smORF length range (5-100 aa) and strong false positive checks to predict smORFs in human gut microbiome data. This results in a vast catalog of smORFs for potential detection in metaproteomic measurements without compromising accuracy or overall proteome coverage. Applied to human gut microbiome samples, this workflow coupled with proteomic validation produced, to our knowledge, the deepest measurements of SEPs, with careful FDR control while utilizing the high-performance Orbitrap Astral mass spectrometer, and outperformed current untargeted and size-exclusion methods^7,54,55^. Importantly, this workflow detects SEPs without experimental enrichment, meaning that SEP presence and abundance is cataloged directly in tandem with the larger proteins, enabling a more comprehensive and bias-free analysis of the untargeted metaproteome as a complete unit. Systematic prediction of smORFs followed by their empirical detection within global metaproteomes from complex gut microbiome samples has been rarely explored, and yet here we demonstrate that it can be achieved with high degree of accuracy. By integrating metagenomic, metatranscriptomic, and metaproteomic data, this framework delivers an expanded multi-omic view of microbial communities, uncovering a previously hidden layer of the metaproteome. Critically, we demonstrate that these smORFs are integral to human gut microbiology, establishing that SEPs can be robustly identified within complex microbial ecosystems. More broadly, this work provides a generalizable framework for multi-omic integration that allows for increased SEP discovery that can be applied across diverse environments, paving the way to elucidate the vast and largely unexplored landscape of SEP biology and its importance in advancing our understanding of microbial and host-associated functions.

## Methods

### Statistics & reproducibility

The sample set was defined by the availability of fecal sample material for proteomic measurements using both instruments, and no statistical method was used to predetermine sample size. The 41 healthy individuals were recruited at specialized sites via different sources as a part of the cross-sectional data of the ExpoBiome cohort described in Hansen et al. (2022)^56^. These samples were used for metagenomics, transcriptomics, and proteomics analyses. De-identification of the data was then done for patient protection. The samples were randomized within metagenomic, transcriptomic, and proteomic preparations to eliminate batch effects.

### Sample collection and handling

The 41 healthy individuals were recruited at specialized sites via different sources as a part of the cross-sectional data of the ExpoBiome cohort described in Hansen et al. (2022)^56^. For each individual, 2-4 vials of stool were collected and immediately flash frozen in liquid nitrogen. Following collection, the samples were shipped to the testing laboratory and stored at −80 °C. Sample and data collection was performed at the two clinical sites in Germany, Charité - Universitätsmedizin Berlin and Paracelsus-Elena Clinic, Kassel.

### Sample aliquoting

Stool samples were initially fractured into smaller pieces (under <190 °C conditions, liquid nitrogen), before being milled into powder and homogenized using a cryomill (6875D Freezer/Mill, SPEX - Instrument Solutions Benelux BV). Milled samples were aliquoted into vials of 150 or 50 mg (+/− 10%) (depending on the intended extraction) under sterile and <190 °C conditions, then stored at −80 °C until use. Each aliquot was manually measured and weighed to allow for relative comparison and normalization during statistical analysis.

### Biomolecular extractions

All intracellular nucleic acids and proteins were extracted from the same 150 mg aliquot. Samples were initially thawed overnight (ON) at −20 °C in 1.5 mL RNA later ICE (Ambion, #4427575), to allow for stabilization of nucleic acids during transition. After incubation ON, samples were processed via a robotics platform: samples were transferred to specialized racks before 3 x 4 mm (Retsch, #R22.455.0001) stainless steel milling balls were added to each vial (at 7 °C). Samples were initially homogenized by shaking at 10 Hz for 2 min (at 4 °C) (Geno/Grinder 2025, SPEX Samples Prep). Vials were centrifuged at 700 x g (4 °C) for 2 min to pellet any physical material, after which the supernatant was transferred to a new vial and rack. Samples were then centrifuged at max speed ( < 4500 x g) for 15 min (4 °C) to pellet the remaining cells and material. The supernatant was automatically removed by the robot after images were taken to calculate the volume of the interphase. Then, 2 x 5 mm (Retsch, #R22.455.0003) and 5 x 2 mm (Retsch, #R22.455.0010) stainless steel balls were added to each vial, in addition to 600 µL of RLT buffer (pre-mixed with 1% β-mercaptoethanol, as per the manufacturer’s instructions). The samples were then quickly vortexed to resuspend the cell pellet, before lysis at 25 Hz for 30 s (at 4 °C) and finally the lysate was then transferred to a 96 DeepWell plate (ThermoFisher #278606) and finally stored at 7 °C before moving onto extractions.

After initial preparation, 1 µL of Riboguard (Lucigen, #RG90910K) was added into the lysate. Samples were then transferred to QIAshredder columns (QIAGEN, #79654) and spun down at 12,000 x g for 3 mins to remove any remaining debris. The flow through was kept and processed at room temperature (RT) using the AllPrep DNA/RNA/Protein mini kit (QIAGEN, #80004), following the manufacturer’s instructions. To ensure suitable drying of protein pellets, and to save time, pellets were dried via SpeedVac (CentriVap Concentrator, Labconco) on a 5–10-minute cycle, run at 4 °C. Pellets were then stored in dry form and no post-processing cleaning or quantification was performed.

DNA and total RNA extracts were also put through a post-processing clean-up protocol to remove any contaminating nucleic acids or chemicals, done before final quantification. The DNA was cleaned using the ZymoResearch DNA clean-up and concentrator kit −5 (ZymoResearch, #D4013), with a minor alteration to the manufacturers protocol; the addition of an RNase incubation before addition of the binding buffer. RNase A (20 µg/µL) (QIAGEN, #19101) was added per sample and incubated at 65 °C for 1 h, before proceeding with the clean-up and concentration. The RNA was cleaned using the ZymoResearch RNA clean-up and concentrator kit −5 (ZymoResearch, #R1013) following the manufacturer’s instructions, including the DNase treatment on column.

After final elution, DNA and total RNA samples were quantified via spectrophotometer (Nanodrop One C, ThermoFisherScientific), fluorometer (Qubit 4, Invitrogen) and quality checked with fragment analyzer (Bioanalyzer 2100, Agilent).

### Sample DNA and RNA sequencing

For metagenomic library preparation, 100 ng of genomic DNA was processed using the xGen DNA Library Preparation Kit (Integrated DNA Technologies, Cat. No. 10009822) using xGen UDI-UMI adapters (Cat. No. 10005903), following the manufacturer’s protocol. The genomic DNA was enzymatically fragmented for 10 min, and DNA libraries were prepared without PCR amplification. The average insert size of libraries was 600 bp. Prepared libraries were quantified using Qubit (DNA HS kit, ThermoFisher) and quality checked with Fragment Analyzer (Agilient).

300 ng of RNA was used for rRNA depletion and library preparation using Illumina stranded total RNA prep with Ribo-Zero Plus microbiome kit (Illumina, 20072063) according to the provided protocol. Prepared libraries were quantified using Qubit 4.0 (DNA HS kit, ThermoFisher) and quality checked with Fragment Analyzer (Agilient).

Sequencing for all libraries was performed at the Luxembourg Center for Systems Biomedicine (LCSB) genomics platform (RRID: SCR_021931) on an Illumina NextSeq2000 instrument using a 2×150 bp read length. The FASTQ files were generated using the onboard Dragon BioIT server version 4.2.7.

### Metagenomics and metatranscriptomics data processing and annotation

Metagenomic and metatranscriptomic data was processed using the integrated meta-omics pipeline (IMP, version 202402_v02)^23,24^. IMP preprocesses, co-assembles the metagenomic and metatrancriptomic reads into contigs, maps the reads, calls single nucleotide polymorphisms, and annotates genes via Mantis^57^. The metagenomic and metatranscriptomic per sample contig statistics can be found in Supplementary Data 12. Mantis is a flexible, consensus-based genome annotation tool that integrates information from multiple reference databases by leveraging both database identifiers and text mining techniques, thus providing several different annotations, including KEGG, COG or CAZy.

### smORF prediction pipeline

To expand the smORFs in the metagenomic and metatranscriptomic data from IMP, a prediction pipeline for smORFs was developed (Supplemental Fig. 1). The MG/MT co-assembled contigs are used by two parallel prediction processes using pyrodigal-gv (version 0.3.2)^14,36^: one targeting smORFs longer than 20 aa (minimum length: 60 nucleotides; maximum overlap: 60 nucleotides), and another for smORF shorter than 20 aa (minimum length: 15 nucleotides; maximum overlap: 15 nucleotides). Each prediction outputs nucleotide and protein FASTA files, along with gene annotation files.

Subsequently, smORFs are filtered to retain only those that meet the following criteria: a maximum length of 100 aa, presence of a ribosome binding site (RBS) motif, and completeness (i.e. presence of start and stop codon (partial=00)). Because smORFs are short and prone to spurious prediction, we applied the completeness check and RBS filter as a conservative strategy to select genuinely translated ORFs. Prodigal learns a genome-specific RBS, starting from a Shine-Dalgarno (SD) assumption and switching to a more general motif finder when SD usage is weak, so it captures both canonical and non-canonical RBS patterns^14^. The filtered smORFs are then dereplicated using MMseqs2 (version 15.6f452; RRID:SCR_022962)^26^ at 100% identity and 100% coverage (-c 1.0 --min-seq-id 1.0 --cov-mode 0 --cluster-mode 0).

To eliminate spurious ORF predictions, the pipeline incorporates three filtering tools. AntiFam (version 8.0)^58^ was applied using PyHMMER with gathering threshold bit score cutoffs to identify spurious protein-coding sequences based on curated hidden Markov models. Infernal (version 1.1.4; RRID:SCR_011809)^59^ was run with cmscan using parameters -rfam --fmt 2 --nohmmonly --cut_tc -g –noali to detect non-coding RNAs against the Rfam database. Aragorn (version 1.2.41.c; RRID:SCR_015974)^60^, was executed twice with parameters –d –w –fasta, using both standard (-gcstd) and bacterial (-gcbact) genetic codes to identify tRNA and tmRNA genes. Predicted smORFs matching any of these databases were excluded from the final per-sample smORF dataset used for the metaproteomics database construction. AntiFam is a curated database of hidden Markov models that are specifically designed to identify spurious protein-coding sequences in genome and metagenome datasets^58^. Infernal is used for searching, aligning, and annotating RNA sequences based on both sequence and secondary structure. It can be used to detect misannotated non-coding RNAs mistakenly predicted as proteins, therefore, being useful to remove false positive entries from the dataset^59^. Aragorn is a tool designed to detect tRNA and tmRNA genes in nucleotide sequences and, on the same line, it can help to remove spurious protein predictions from a database^60^.

The predicted smORFs taxonomic annotations are obtained by using MMseqs2 (version 15.6f452; RRID:SCR_022962) with GTDB (version 10-RS226). The parameters used are easy-taxonomy --tax-lineage 1 -v 1 --report-mode 1. The functional annotations are obtained via Mantis using the default parameters (version 1.5.5; RRID:SCR_021001)^57^. Mantis uses five databases: KOfam, Pfam, eggNOG, NCBI protein family models (NPFM) and TIGRfams^57^.

We benchmarked our smORF prediction pipeline against smORFinder^5^ using *Escherichia coli* K-12 substrain MG1655 (NC_000913.3), which EcoCyc reports as having 536 validated small proteins ≤100 aa (164 ≤ 50aa)^25^. Predictions from both tools were validated against EcoCyc proteins using MMseqs2 easy-search at 100% identity and 90% coverage to account for boundary differences^26^. We also ran our study’s co-assembled contigs on smORFinder and compared the results to ours using MMSeqs2 (100% identity 90% coverage)^26^. GMSC-mapper was run on both datasets to obtain homologs to a published smORF catalog^8,32^. Full benchmarking details, parameters, and results are in Supplemental Method 1.

### Metaproteomics database construction

To construct a high-confidence protein database for the metaproteomic searches (Supplemental Fig. 2), we first retrieved the IMP-predicted protein sequences and modified their FASTA headers to include both the corresponding SEP ID and the IMP identifier. A similar procedure was applied to the SEPs predicted by the smORF prediction pipeline.

We then downloaded the human reference proteome from NCBI (version 2025-06-10; GRCh38.p14; RRID:SCR_006472)^27^, removed duplicate entries with MMseqs2 easy-cluster with the parameters -c 1.0 --min-seq-id 1.0 --cov-mode 0 --cluster-mode 0 (version 15.6f452; RRID:SCR_022962), and updated the FASTA headers to include the SEP ID and a human-specific identifier. Additionally, we incorporated the common Repository of Adventitious Proteins (cRAP) database (version 2025-06-14; RRID:SCR_018187)^28^, obtained from the Cambridge Center for Proteomics (CCP), and similarly annotated its entries.

We concatenated the IMP and smORF protein datasets for each sample and removed redundant sequences using MMseqs2 easy-cluster with the parameters -c 1.0 --min-seq-id 1.0 --cov-mode 0 --cluster-mode 0 (version 15.6f452; RRID:SCR_022962). In parallel, we merged the human and cRAP database and eliminated duplicates using the same MMseqs2 parameters (version 15.6f452; RRID:SCR_022962). To ensure the removal of potential contaminants, we compared the IMP-smORF dataset against the combined human-cRAP database and excluded any sequences that were identical to known human or contaminant proteins using MMseqs2 (version 15.6f452; RRID:SCR_022962) as previously reported.

Finally, we concatenated all sample-specific protein datasets and performed clustering at 100% identity and 100% coverage using MMseqs2 easy-cluster with the parameters -c 1.0 --min-seq-id 1.0 --cov-mode 0 --cluster-mode 0 (version 15.6f452; RRID:SCR_022962). This allowed us to identify representative sequences and their corresponding cluster members. The SEP IDs of all cluster members were then updated to match those of their representative sequences, resulting in the final per-sample protein databases. This pipeline ensures that the resulting IMP-smORF protein datasets are free from human and common contaminant sequences, while still retaining the identifiable entries for human and contaminant proteins through their annotated FASTA headers.

A human smORF catalog (1509 from reference proteome^27^ + 7554 from Martinez et al.^29^ + 150,802 from OpenProt^30^; final dereplicated: 157,901) was concatenated with microbial smORFs from our database and clustered via MMseqs2 easy-cluster at different thresholds^26^. Mixed clusters ( ≥ 1 human + 1 microbial smORF) were identified; human annotations were obtained from OpenProt^30^. Full catalog construction, parameters and results are found in Supplemental Method 2.

Non-redundant smORFs from our database were queried against GMSC v1.0 using GMSC-mapper^8,32^. Habitat, taxonomy, quality, and CDD annotations transferred from hits. Full details in Supplemental Method 3.

The metaproteomic database was clustered with MMseqs2 easy-cluster at 95% identity and 95% coverage as used in previous microbial smORF studies to obtain biological interpretation^26^. Full workflow in Supplemental Method 4.

### Sample preparation & LC-MS/MS analysis

The intracellular proteins were extracted utilizing the AllPrep DNA/RNA/ Protein kit from (QIAGEN). Extracted protein samples were shipped using dry ice then kept at −80 °C till use. Proteins extracts were re-solubilized in Tris solution with 4% sodium dodecyl sulfate and 10 mM Dithiothreitol and heated for 10 min at 90 °C. 30 mM iodoacetamide was then added and incubated for disulfide bond reduction. After this, samples were then prepped using the Protein Aggregation Capture (PAC) bacterial method (going through 2 series of bead aggregation and washing to ensure accurate quantification)^61^. Proteins were then digested twice (3 h and overnight) using trypsin at a 1:75ug ratio and 0.5% formic acid was added for acidification. Peptides were filtered using a 10 kDa MWCO filter (Vivaspin500 PES; Sartorius).

All samples were run using two different reverse-phase LC-MS/MS set-ups: 1) 2ug peptide load run on a Vanquish Neo HPLC system coupled to an Orbitrap Astral mass-spectrometer using a C18 column (Thermo Scientific Easy-Spray™ PepMap™ Neo 2 μm C18 75 μm X 150 mm)) for a DIA 60-minute organic gradien,t or 2) 5ug peptide load run on a Vanquish HPLC coupled to a Q Exactive Plus mass spectrometer using a C18 column (5 μm Kinetex; Phenomenex, 100 μm ID x 150 mm) made in-house for a DDA 180-minute organic gradient.

Both sets of raw files were then searched against the per-sample databases with smORF predictions that are described above. Searches were done using Proteome Discoverer (version 3.2, Thermo Scientific) with Chimerys. Quantification for DIA samples was done with MS2 Apex Quantification, and MS1 AUC (area under the curve) quantification for DDA samples. Both searched for fully-tryptic peptides with a 7 aa minimum peptide length, 2 ppm mass tolerance, 2 max missed cleavages, and 1% peptide level FDR. Oxidation of methionine was set as a dynamic modification and cysteine carbamidomethylation was set as a static modification. In the search databases, 98.21% of small proteins ( ≥ 100 aa) contained at least one tryptic peptide, because of this, searches were kept to fully-tryptic peptides, and not expanded for semi or non-tryptic peptides, in order to limit search database size which in turn can inflate false positive identifications.

Though the global searches, including the SEP identifications, were controlled at a 1% peptide level FDR, subsets of proteomes can carry differing FDRs^62^. In order to interrogate the composition of the SEP detection, a SEP-specific false discovery rate (FDR) at the peptide level was calculated and evaluated with an entrapment database. The SEP-specific false discovery rate (FDR) were calculated first with an entrapment database comprised of marine archaea sequences^32^, then secondly, an entrapment database created from compiling the SEP database sequences and randomly shuffling each sequence. The creation of these databases and the calculations are further detailed in Supplemental Method 5. These SEP-specific FDR calculations were done for 12 randomly selected samples, for both the Orbitrap Astral platform (Fig. 3A), and the Q Exactive Plus instrument runs (Supplemental Fig. 7A). While the SEP-specific FDR exceeded the conventional 1% threshold, it remained near the widely accepted more liberal 5% peptide FDR cutoff commonly used in proteomic studies^44,63–67^. The intent of reporting the smORF-specific FDR values is to transparently illustrate and emphasize this phenomenon when expanding search spaces in any manner, including but not exclusive to small protein discovery. The SEP-specific estimates simply provide additional context for interpreting small protein identifications and do not alter the primary filtering criteria used in the study.

### Quantification of proteins, functions, and taxa

Peptide tables, resulting from Proteome Discoverer raw file searches, were first filtered to remove those which could be generated by proteins in the cRAP contaminants database^28^. Raw peptide intensities were midmean (the mean of values in the interquartile range) centered. Proteins and eggNOG ortholog groups were quantified by summing over unique peptides. Taxa (extrapolated from the database) were quantified by summing the intensities of all peptides that uniquely mapped to each taxon. Protein and taxonomy quantification are further explained in Supplemental Method 6.

### Ethics declaration

This study was done using stool samples from human participants. Ethical approval was obtained to plan and conduct the trial from the institutional review board of the Charité-Universitätsmedizin Berlin (EA1/204/19), the ethics committee of the state medical association (Landesärztekammer) of Hessen (2021-2230-zvBO) and the Ethics Review Panel (ERP) of the University of Luxembourg (ERP 21-001 A ExpoBiome).

### Reporting summary

Further information on research design is available in the Nature Portfolio Reporting Summary linked to this article.

## Supplementary information

Supplemental Information

Description of Additional Supplementary Files

Supplementary Data 1. Benchmarking results

Supplementary Data 2. Unique and shared predicted smORFs between samples table

Supplementary Data 3. Predicted smORFs sequence length distribution table

Supplementary Data 4. Human-microbial homology analysis results at different identities and coverages

Supplementary Data 5. Clustering metrics

Supplementary Data 6. Top taxa prevalence in each taxonomic rank

Supplementary Data 7. Core smORFs taxonomic annotation

Supplementary Data 8. Functional annotation of core smORFs

Supplementary Data 9. Detected Proteins including SEPs (Orbitrap Astral and Q Exactive Plus)

Supplementary Data 10. SEP summed abundance to taxonomy/annotation

Supplementary Data 11. Global summed abundance to taxonomy/annotation

Supplementary Data 12. Summary per sample contig statistics

Reporting Summary

Transparent Peer Review file

## Data Availability

The metagenomic and metatranscriptomic files generated in this study are available in the European Read Archive ENA under Project ID PRJEB97797. The metaproteomic raw files, and corresponding search results generated in this study are deposited in the Proteome XChange Consortium via the MassIVE repository under the accession ID MSV000099334 [10.25345/C50V89W6F].

## Code availability

The smORFs prediction pipeline and the database creation pipeline are available for download at https://gitlab.com/uniluxembourg/lcsb/systems-ecology/smorf_methods_paper.
