# Supplementary material for: High-resolution multi-omics enhances prediction and detection of smORF-encoded proteins in the human gut microbiome
Source: Nat Commun. 2026 May 9;17:6296. doi: 10.1038/s41467-026-72762-5 (PMC13376575; doi:10.1038/s41467-026-72762-5)
Supplement: Supplementary file 1 — Supplemental Information [file 41467_2026_72762_MOESM1_ESM.pdf]

Supplemental Information

This document includes all Supplemental Text and Figures in order of appearance.

All citations can be found within the main text.

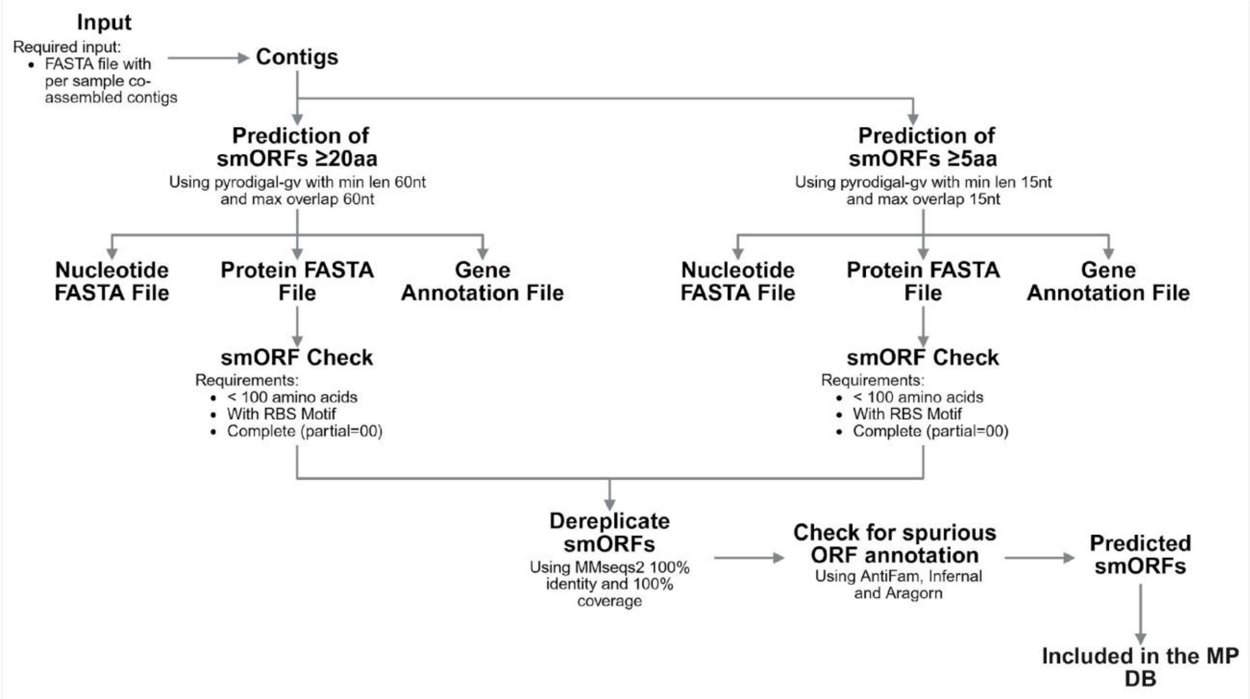

Supplemental Figure 1. Overview of the small open reading frames (smORFs) prediction pipeline. Comprehensive pipeline for predicting and annotating smORFs from nucleotide FASTA files containing per-sample co-assembled (i.e. MG and MT) contigs. The pipeline is divided into two branches based on smORF length: those greater than 20 amino acids and those shorter. Each branch includes prediction using pyrodigal-gv, smORF checks based on specific criteria and dereplication using MMseqs2 100% identity and 100% coverage. Additional steps include filtering spurious ORFs using AntiFam, Infernal and Aragorn, which lead to the final predicted smORFs per sample database, which is then included in the final metaproteomics per sample database.

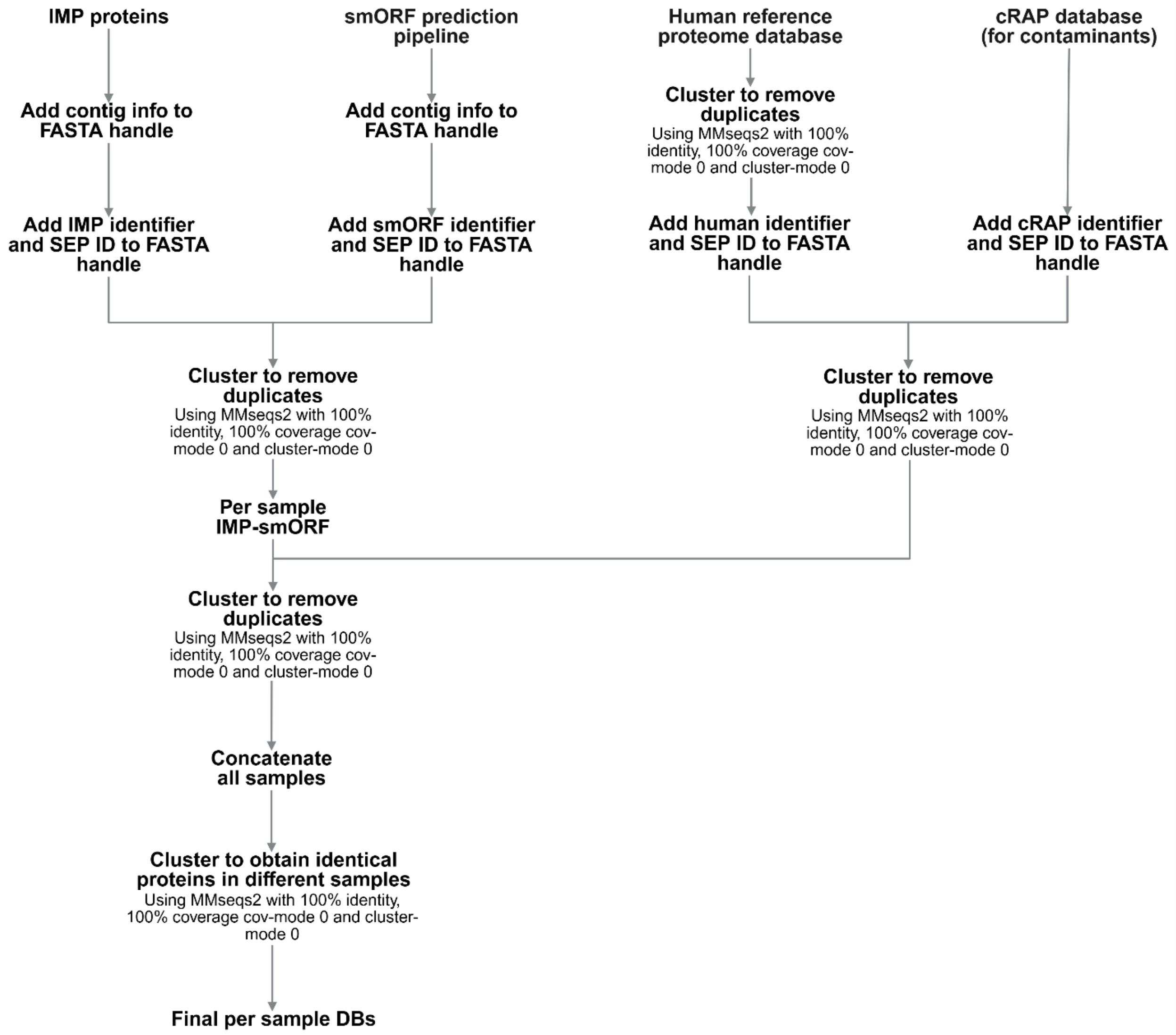

Supplemental Figure 2. Overview of the metaproteomic database creation process. Outline of the step-by-step workflow used to construct sample-specific protein databases by integrating proteins from multiple sources: IMP-derived proteins, smORFs predicted via our dedicated pipeline, the human reference proteome, and the cRAP contaminant database. Each source undergoes preprocessing steps including the addition of contig information, unique identifiers, and SEP IDs. Redundancy is removed through iterative clustering using MMseqs2 at 100% identity and 100% coverage. After source-specific processing, proteins are merged, clustered across samples to identify shared sequences, and compiled into final per-sample protein databases for downstream metaproteomic analysis.

Supplemental Note 1:

To evaluate the performance of our prediction pipeline, we benchmarked our tool against smORFinder, a command-line tool used to identify and annotate small proteins that are 50 amino acids or less in genomes and metagenomes. We used *E. coli* K-12 substrain MG1655, a bacterial strain with 536 validated small proteins in EcoCyc, 164 of them being ≤50 amino acids in length. Based on this genome, smORFinder predicted 17 smORFs ≤50 amino acids (**Supplemental Table 1**). On the same dataset, our pipeline predicted 450 smORFs up to 100 amino acids, including 91 smORFs ≤50 amino acids (**Supplemental Table 1**). To validate the results, we compared the predictions from both tools against the EcoCyc-curated small proteins using MMseqs2. Using stringent criteria (100% identity and 100% coverage), smORFinder recovered 0/164 (0%) of the EcoCyc-annotated proteins ≤50 amino acids, while our pipeline recovered 41/164 (25%) of proteins ≤50 amino acids and 343/536 (64%) of all small proteins up to 100 amino acids (**Supplemental Table 1**). When we relaxed the coverage threshold to 90% to account for minor annotation boundary differences, smORFinder recovered 16/164 (9.8%) and our pipeline recovered 42/164 (25.6%) of proteins ≤50 amino acids and 342/536 (63.8%) of all small proteins up to 100 amino acids (**Supplemental Table 1**). Under both validation criteria, our pipeline outperformed smORFinder in recovering validated small proteins.

In addition, we ran smORFinder on our co-assembled contigs to assess overlap with our pipeline predictions. SmORFinder predicted 77,027 non-redundant smORFs of ≤50 amino acids, whereas our pipeline predicted 524,743 smORFs. Of the smORFs predicted by smORFinder, 76,261 smORFs were also predicted by our prediction pipeline. We then evaluated proteomic support for the smORFinder prediction: 628 smORFs (32.7%) were also detected in our metaproteomics-validated dataset, which contains 1,921 SEPs overall. Finally, we mapped smORFs from both approaches to the Global Microbial smORF Catalogue (GMSC). Of the smORFs predicted by our pipeline, 448,530 (85.47%) had homologs in GMSC, compared with 71,542 (92.88%) of smORFinder-predicted smORFs.

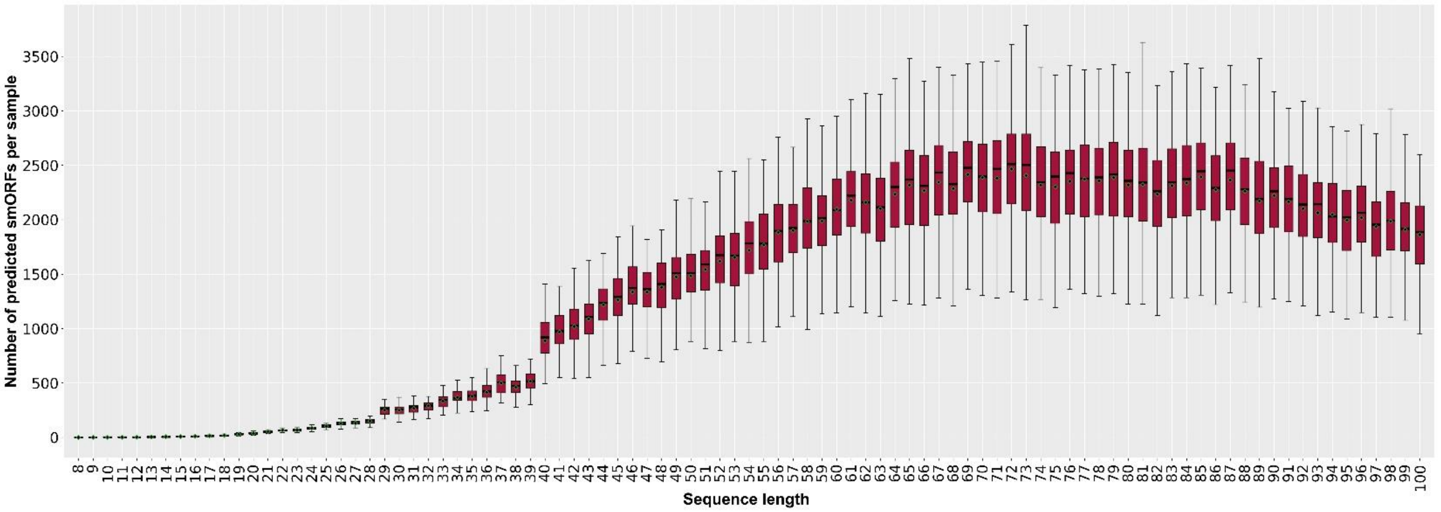

Supplemental Figure 3 Sequence length distribution plot of predicted smORFs, without bins. For each amino acid length, box plots show the distribution of the number of predicted smORFs per sample across n = 41 samples. Box plots show the median (center line), interquartile range (box; 25th–75th percentile), and whiskers extending to the most extreme values within 1.5 × IQR; outliers are not shown. The black diamond indicates the mean.

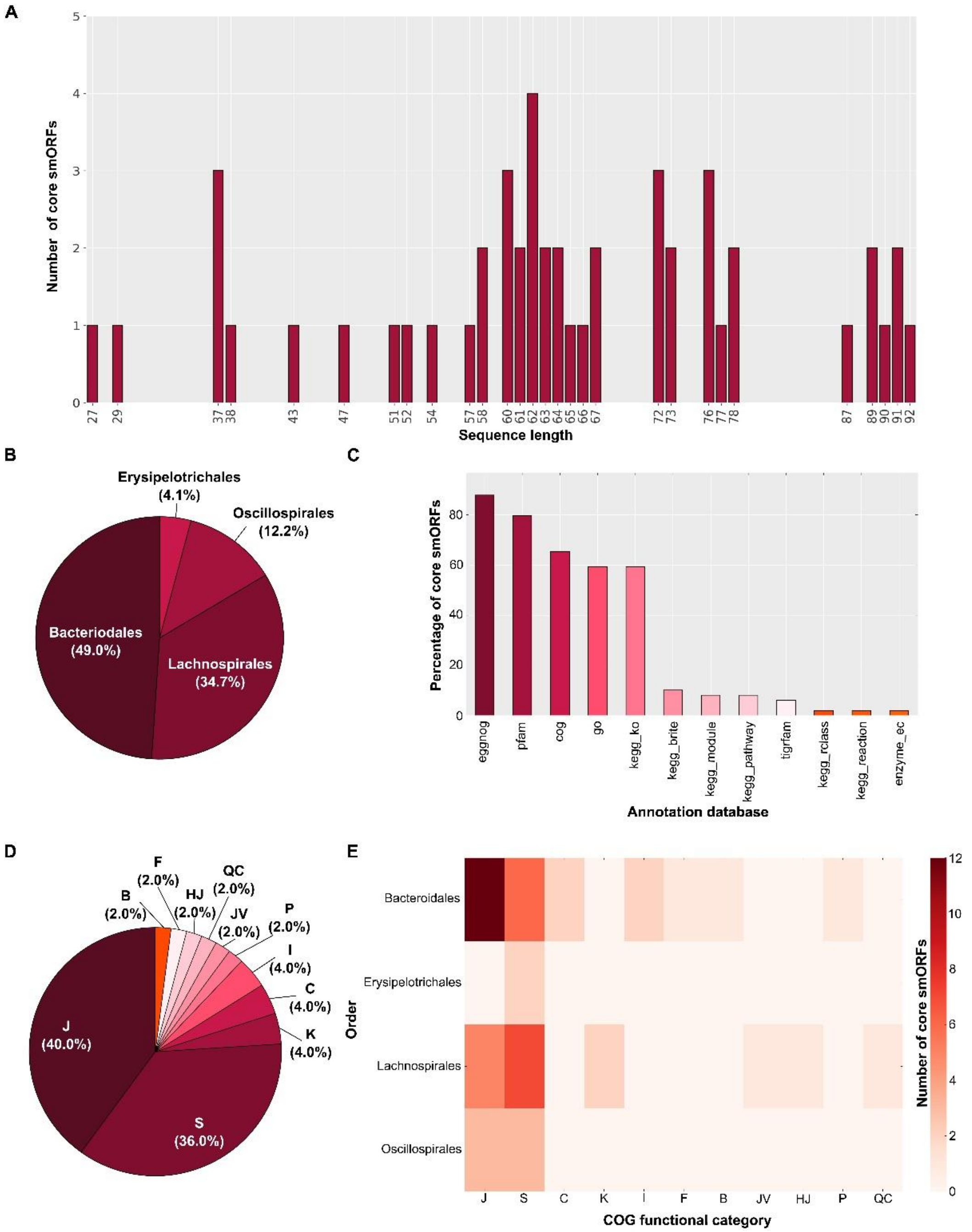

Supplemental Figure 4. Core smORFs A) Sequence length distribution of the core smORFs. **B)** Taxonomical profiles of the core smORFs at the order level. At the order level all the smORFs are classified to the orders Bacteriodales, Lachnospirales, Oscillospiralles or Erysipelotrichales. **C)** Annotation coverage for each Mantis database for core smORFs. Broader databases, such as EGGNOG or Pfam, have a higher annotation proportion, in contrast to more specific databases, such as kegg_reaction. Mantis databases without any core smORF annotated are not shown. **D)** Pie chart of the COG functional categories for the core smORFs. The functions reported are J: translation, ribosomal structure and biogenesis, S: function unknown, K: transcription, C: energy production and conversion, I: lipid transport and metabolism, P: inorganic ion transport and metabolism, QC: secondary metabolites and energy production, JV: translation and defense mechanisms, HJ: coenzyme metabolism and translation, F: nucleotide transport and metabolism and B: chromatin structure and dynamics. **E)** Heatmap of the COG functional categories of the core smORFs for each order. The heatmap reveals that most of the Bacteroidales smORFs have the function J. Something similar can be seen in Lachnospirales and Oscillospirales. On the other hand, the smORFs belonging to Erysipelotrichales have an unknown function (S).

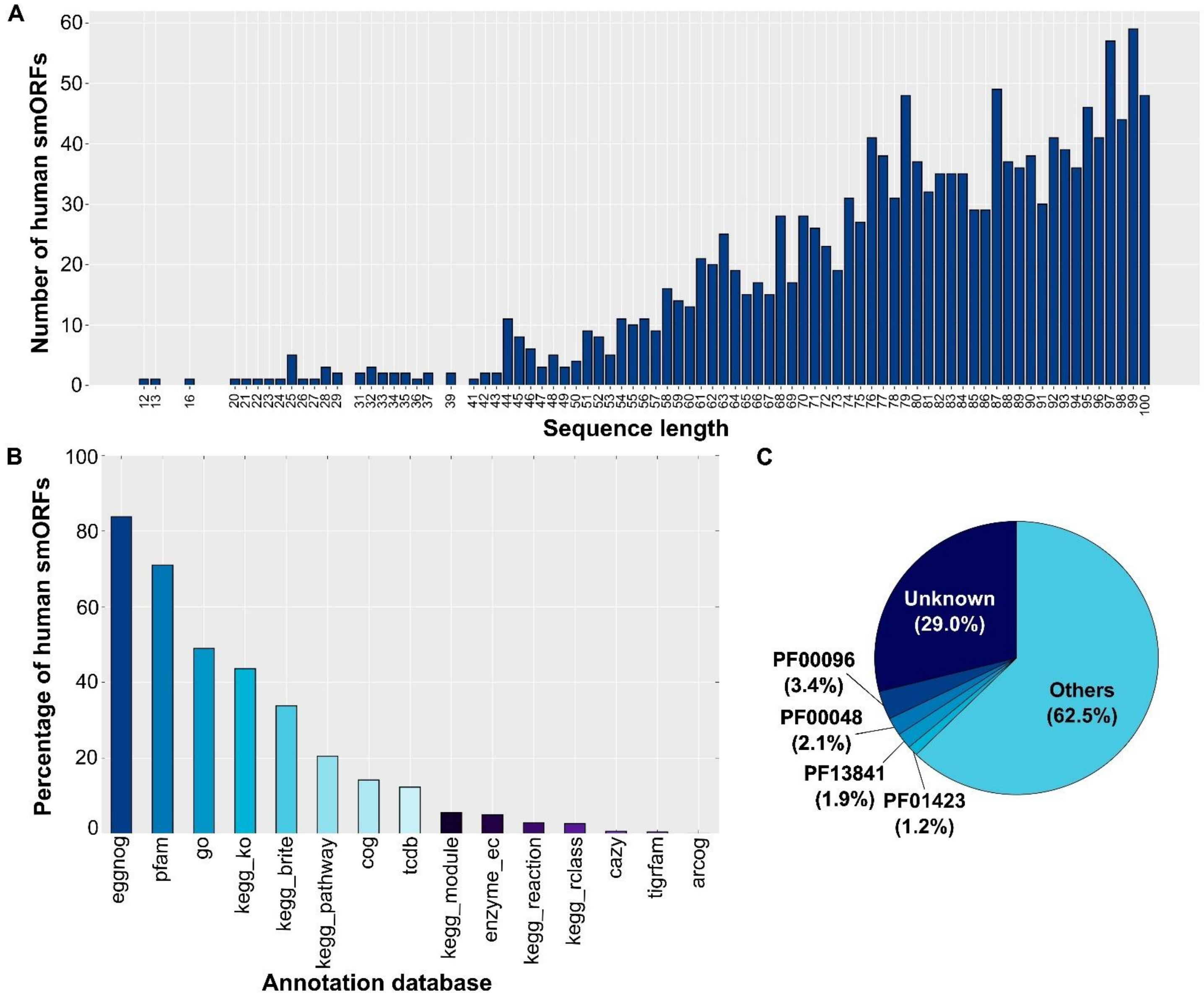

Supplemental Figure 5. Human smORFs A) Sequence length distribution of the human smORFs. **B)** Annotation coverage for each Mantis database for human smORFs. Broader databases, such as eggNOG or Pfam, have a higher annotation rate, in contrast to more specific databases, such as ArCOG or TIGRFAM. **C)** Pie chart of the Pfam functional annotation. A total of 1072 human smORFs could be annotated. The most common domains were PF00096 (zinc finger, C2H2), PF00048 (small cytokines, interleukin-8 like), PF13841 (beta defensin) and PF01423 (LSM domain). The rest of the Pfam domains are assigned to less than 1% of the smORFs and are grouped together in "Others".

Supplemental Note 2:

We used a comprehensive human smORF catalogue built from three sources: the smORFs from human reference proteome, the 7,554 human smORFs described in Martinez et. al., and the 150,802 human smORFs supported by at least two unique peptides from OpenProt. To evaluate homology, these human smORFs were clustered together with the microbial smORFs at different identities and coverages using MMseqs2. At stringent thresholds (100% identity / 100% coverage and 95% identity / 95% coverage), no mixed clusters containing both human and microbial smORFs were detected (**Supplemental Table 4**). At more relaxed thresholds (90% identity / 80% coverage), more permissive than the parameters used in microbiome smORF studies, only 4 human smORFs clustered with microbial smORFs (**Supplemental Table 4**). These human smORFs map to core, widely conserved protein families: a RAB2B-associated member of the Ras oncogene family, two histone H3 variants, and an alternative ubiquitin-like protein. Having confirmed minimal overlap between human and microbial smORFs, we focused our subsequent analyses on microbial smORFs/SEPs, as human smORFs were not the primary focus of this study.

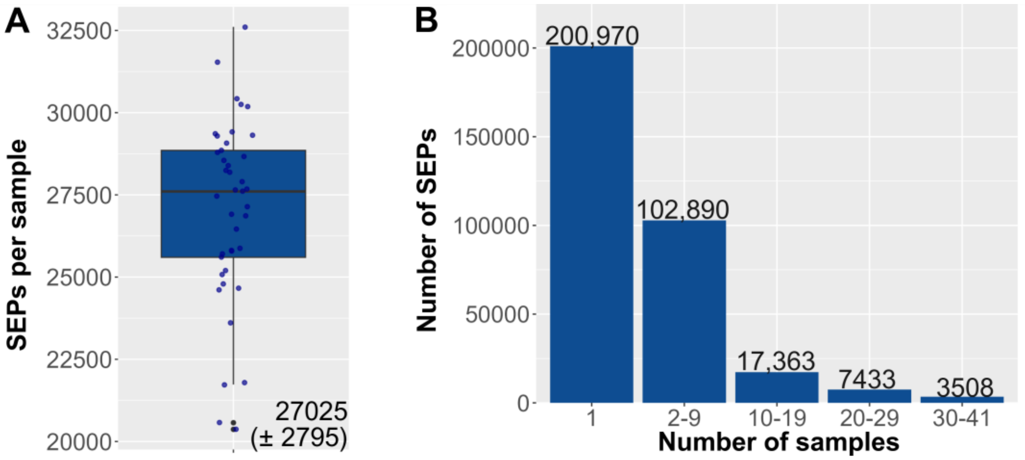

Supplemental Figure 6 Detection of proteins, including SEPs, in the Global Proteome. A) Distribution of the total proteins detected per sample. Box plots show individual data points over the median (center line), interquartile range (box; 25th–75th percentile), and whiskers extending to values within 1.5 × IQR. Mean values ± STD are reported in the bottom right corner. **B)** Protein presence across samples in 1, 2-9, 10-19, 20-29, or 30-41 samples.

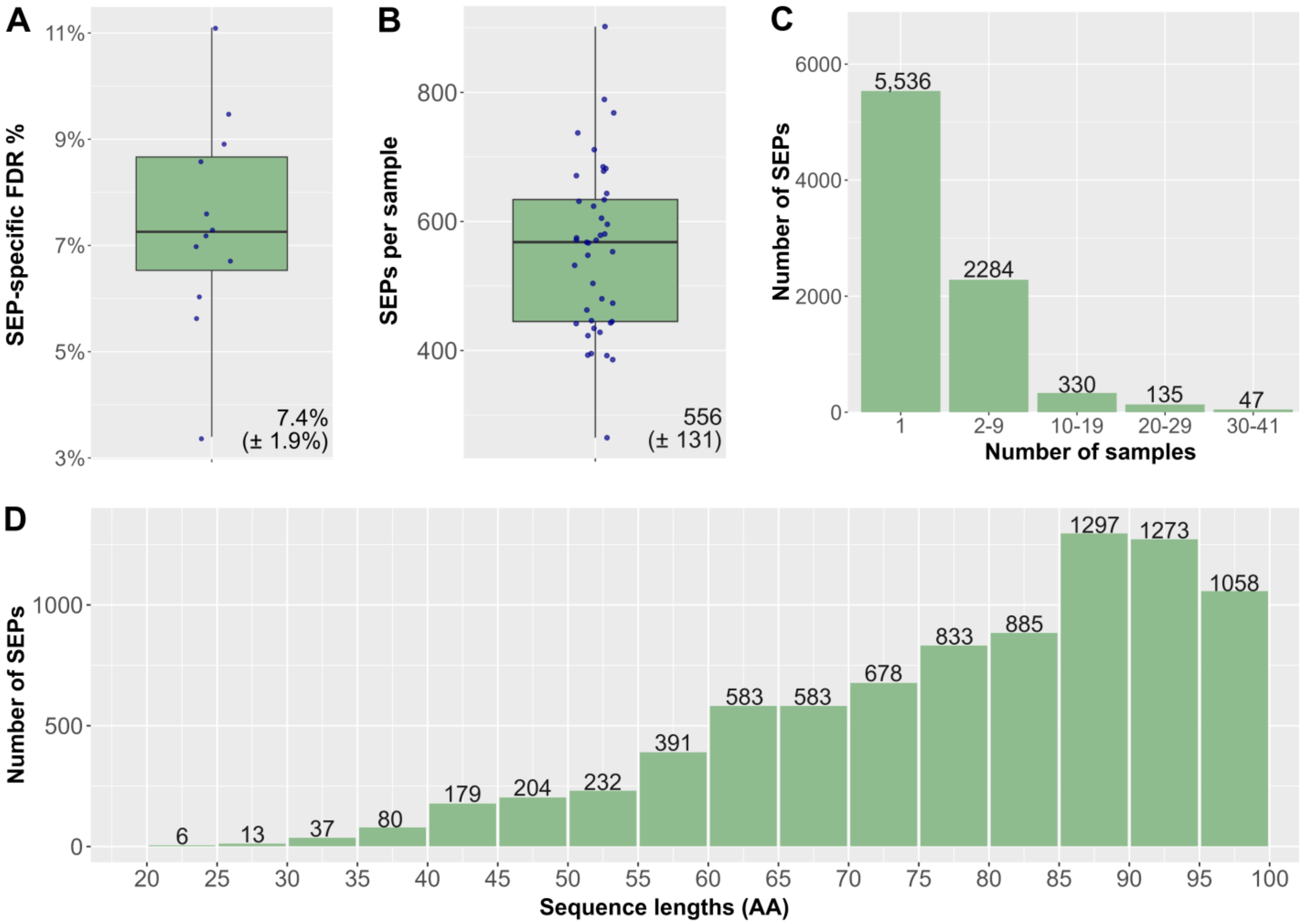

Supplemental Figure 7. Detection of SEPs using the Q Exactive Plus instrument A) Distribution of upper-bound SEP-specific (peptide level) FDRs calculated with SEP-specific entrapment database (n=12). Box plots show individual data points over the median (center line), interquartile range (box; 25th–75th percentile), and whiskers extending to values within 1.5 × IQR. Mean values ± STD are reported in the bottom right corner. **B)** Distribution of the total SEPs detected per sample (n=41). Box plots show individual data points over the median (center line), interquartile range (box; 25th–75th percentile), and whiskers extending to values within 1.5 × IQR. Mean values ± STD are reported in the bottom right corner. **C)** SEP presence across samples in 1, 2-9, 10-19, 20-29, or 30-41 samples. **D)** Amount of detected SEPs across sequence length distribution (bin=5) for all SEPs detected in study. Counts for contributing SEPs are listed above each bar in C/ B.

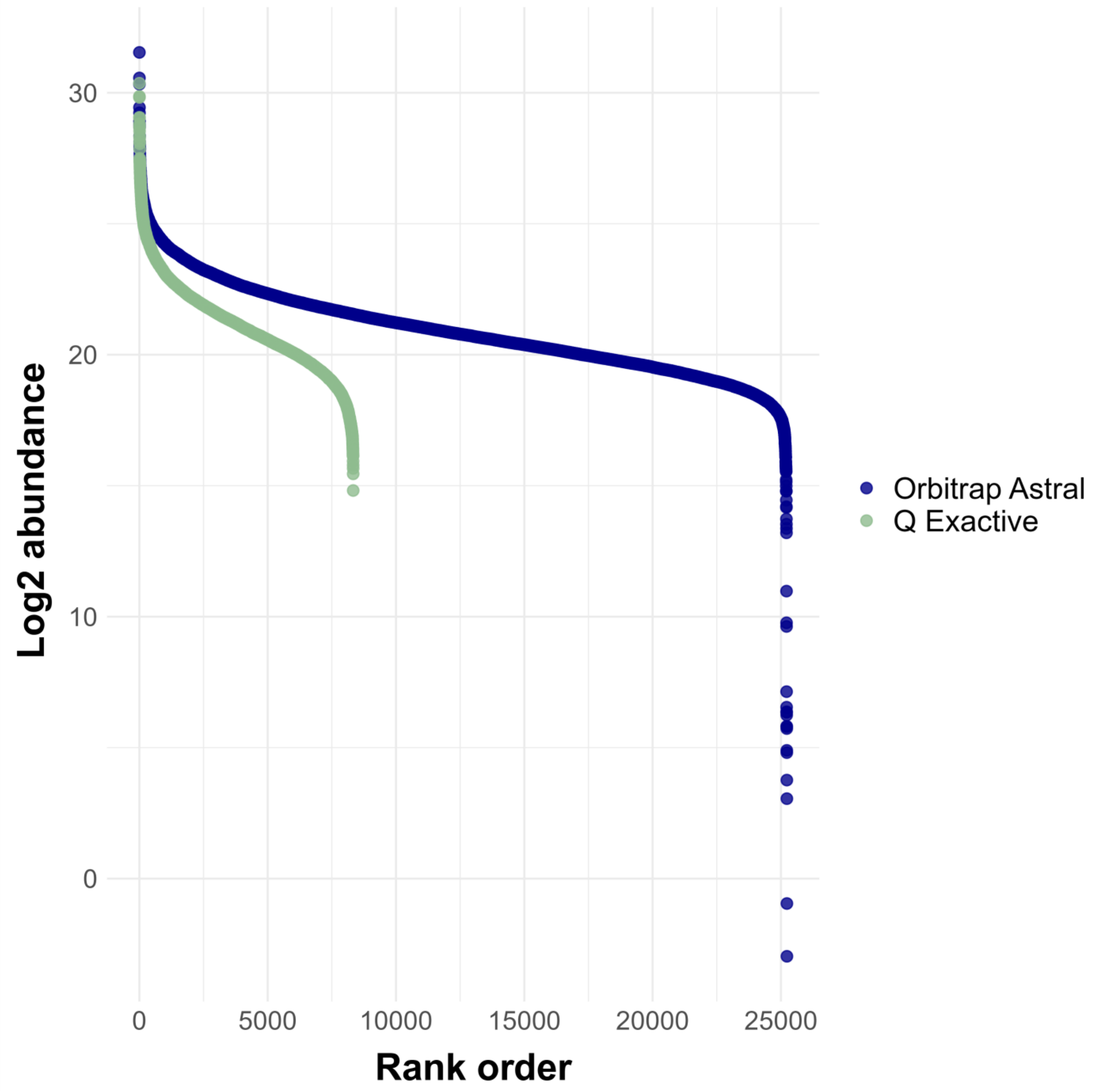

Supplemental Figure 8. Instrument comparison of the ranked detected SEPs A rank abundance graph of the SEPs detected from the DIA Orbitrap Astral Data and the DDA Q Exactive Plus data. The green line represents data from the Q Exactive and Blue line represents data from the Orbitrap Astral.

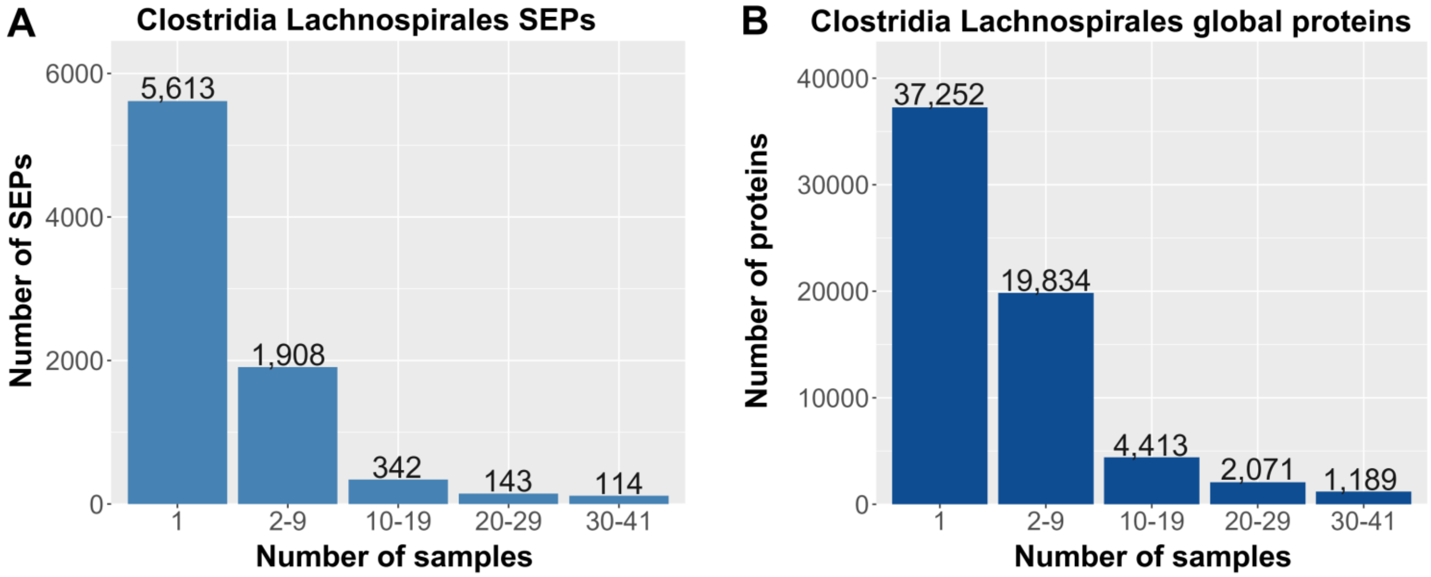

Supplemental Figure 9 Frequency of Proteins and SEPs detected for Clostridia Lachnospirales. A) Lachnospirales SEPs detected in 1, 2-9, 10-19, 20-29, or 30-41 samples. **B)** Lachnospirales proteins detected in 1, 2-9, 10-19,20-29, or 30-41 samples.

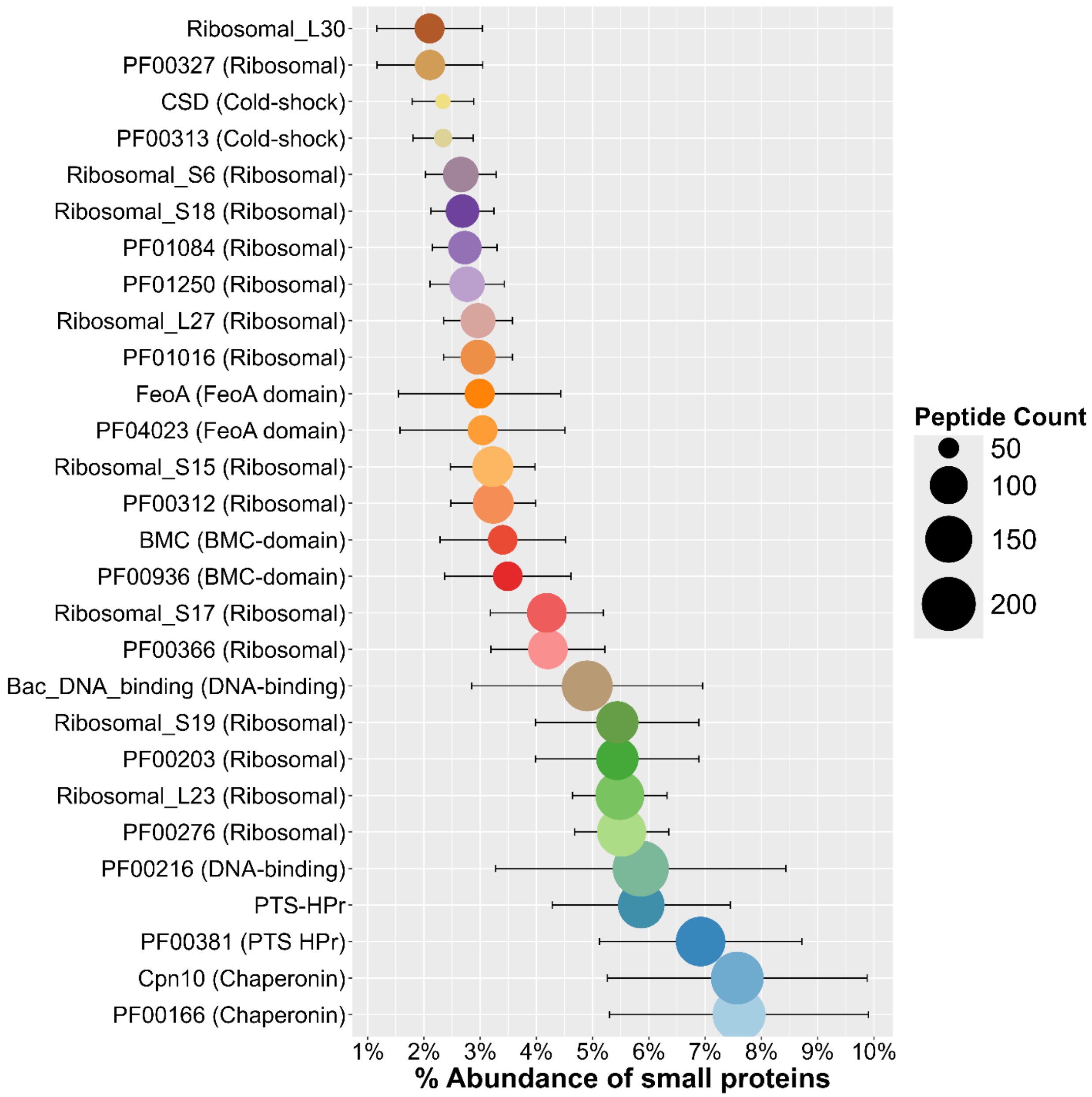

Supplemental Figure 10. Top Pfam functional categories via percentage of SEP abundance Pfams that have over 2% of the total SEP abundance mapped to an ID are shown. Pfam codes are followed by a brief description in parentheses where needed. Dot size represents the average amount of peptides represented (sample mean). Abundance percentage was calculated by summing SEP peptide abundances mapping to each functional annotation and dividing by total SEP peptide abundance. Percentages were then averaged across samples (error bars ± STD).

Supplemental Discussion 1:

Although our pipeline was developed and tuned for large-scale smORF discovery in complex gut metagenomic assemblies rather than for single, well-annotated isolate genomes, we evaluated the performance on *E. coli K-12 substrain MG1655* because it provides an unusually well-curated reference set of small proteins, enabling an objective recovery-based benchmark that is currently difficult to replicate for human gut metagenomic datasets. Our pipeline substantially outperformed smORFinder under both stringent (100% identity and 100% coverage) and relaxed (100% identity and 90% coverage) validation criteria. Notably, our pipeline showed consistent recovery rates across both thresholds, indicating accurate prediction of sequence boundaries, whereas smORFinder only recovered matches when allowing for annotation boundary differences. Even though EcoCyc provides an unusually well-curated reference set for *E. coli* K-12 substrain MG1655 small proteins, complete recovery of all curated smORFs is not expected for any single prediction strategy. Short genes often lack strong gene-like signals and can exhibit non-canonical architectures, making it difficult to predict them with one "standard" prediction pipeline. To complement the isolate-genome benchmark, we also compared smORFinder with our pipeline on the co-assembled contigs used throughout this study. Despite large differences in the number of predicted smORFs, most smORFinder predictions overlapped with our call set under a more relaxed sequence-based matching criteria (100% identity and 90% coverage). At the same time, only a subset of smORFinder predictions were supported by metaproteomics in our dataset.

Supplemental Discussion 2

To evaluate whether the size of the human smORF database impacted the results, we expanded the human smORF catalogue used in the metaproteomics database to include a larger set of experimentally validated human smORFs. The human-microbial smORFs homology analysis revealed only a very small number of conserved human-microbial smORFs, indicating that cross-kingdom sequence homology at the smORF level is rare and largely restricted to core, widely conserved protein families. The few overlapping smORFs corresponded to histone-like proteins, ubiquitin-like systems, and Ras-superfamily small GTPases, all of which have established bacterial counterparts, consistent with their fundamental roles in genome organization, protein homeostasis, and signaling across diverse domains of life. This pattern suggests that, beyond these conserved molecules, most human and microbial smORFs are likely lineage-specific, supporting a model in which smORF repertoires evolve rapidly and may encode host- or niche-adapted functions rather than broadly conserved housekeeping roles. Importantly, the lack of of shared smORFs also implies that restricting the metaproteomic database to the 1,509 human smORFs from the human reference proteome is unlikely to introduce substantial misassignment of human spectra to microbial peptides, as the detectable sequence space shared between human and microbial smORFs appears to be extremely limited.

Supplemental Method 1:

We benchmarked our smORF prediction pipeline against smORFinder, a command-line tool for identifying and annotating small proteins up to 50 amino acids in genomes and metagenomes. Benchmarking was performed on *Escherichia coli* K-12 substrain MG1655 (NCBI Reference Sequence: NC_000913.3). EcoCyc, a database specialized on *E. coli K-12* substrain MG1655 that describes its genome, metabolic pathways and regulatory network, reports 536 validated small proteins of ≤100 amino acids in this strain, including 164 proteins of ≤50 amino acids. Both the genome and the coding proteins for *Escherichia coli* K-12 substrain MG1655 were extracted from NCBI using the reference sequence reported in EcoCyc. The genomic FASTA file was used to predict the smORFs, while the protein FASTA file was used to validate the results.

SmORFinder was executed following the repository documentation using "smorf single fasta_file.fasta". Our pipeline was run on the same input dataset and generates predictions up to 100 amino acids. For comparability with smORFinder, we modified the –p parameter in our prediction rule from meta to single, as we are predicting smORFs on a single genome. In addition, we only retained predictions of less than 50 amino acids prior to validation.

Predicted smORFs from both tools were validated against the 536 EcoCyc small proteins using MMseqs2 easy-search. Initial validation required 100% sequence identity and 100% coverage (--min-seq-id 1.0 -c 1.0 --cov-mode 0). Due to potential annotation boundary differences between predicted and reference sequences, we relaxed the coverage threshold to 90% (-c 0.9) while maintaining the identity in aligned regions to account for minor variations in start/stop codon predictions that do not reflect biological differences.

To compare smORFinder predictions with those from our pipeline on our study dataset, smORFinder was run in metagenomics mode on the same co-assembled contigs used as input to our prediction pipeline using "smorf meta fasta_file.fasta". To quantify the overlap between the two prediction pipelines, we performed sequence-based matching with MMSeqs2 (--min-seq-id 1.0 -c 0.9 --cov-mode 0).

Supplemental Method 2:

To assess potential homology between human and microbial smORFs, a comprehensive human smORF catalogue was constructed from three sources: the 1,509 smORFs from the human reference proteome, the 7,554 human smORFs reported by Martinez et al., and the 150,802 human smORFs supported by at least two unique peptides in OpenProt. The human smORF catalogue was dereplicated to remove redundant sequences, leading to a final set of 157,901 human smORFs. Human and microbial smORF amino acid sequences were concatenated and clustered with MMSeqs2 (version 15.6f452; RRID:SCR_022962) easy-cluster with parameters --cov-mode 0 and --cluster-mode 0 at multiple sequence identity and coverage thresholds (100% identity / 100% coverage; 95% identity / 95% coverage; 90% identity / 90% coverage; and 90% identity / 80% coverage). Clusters were classified as mixed if they contained at least one human and one microbial smORF. The functional annotation of the human smORFs was obtained from OpenProt.

Supplemental Method 3:

Non-redundant predicted smORFs from our prediction pipeline as well as from smORFinder were queried against the GMSC using GMSC-mapper. A DIAMOND-formatted index text of the current GMSC release (GMSC10.90AA.faa.gz, corresponding to v1.0) was built using ‘gmsc-mapper createdb’, and alignments were performed with DIAMOND (default in GMSC-mapper). Default mapping parameters were used, and smORFs with hits passing the thresholds were considered represented in GMSC. Habitat, taxonomy, quality, and CDD annotations were then transferred from the matched GMSC homologs as reported by GMSC-mapper output tables.

Supplemental Method 4:

To facilitate biological interpretation of metaproteomic results, the comprehensive protein sequence database was clustered to group homologous sequences. Protein sequences were clustered using MMseqs2 easy-cluster workflow with parameters set to 95% minimum sequence identity (-min-seq-id 0.95) and 95% coverage (-c 0.95), as used previously in similar studies. Clustering was performed using coverage mode 0 (--cov-mode 0) and cluster mode 0 (--cluster-mode 0).

Supplemental Method 5:

To evaluate the accuracy of the SEP detection, a SEP-specific false discovery rate (FDR) at the peptide level was calculated using an entrapment database approach. Twelve samples were randomly selected and searched against a combined database consisting of the sample-specific database and an entrapment database. This analysis was first done with an entrapment database curated from marine archaea sequences unrelated to the gut microbiome. The second entrapment database was created from compiling the SEP database sequences and randomly shuffling each sequence; this was done to maintain the amino acid distributions from the original sample databases. Each entrapment database was filtered to include only sequences with ≤60% similarity to all sample-specific gut microbiome databases before concatenation at a 1:1 ratio of predicted SEPs to SEP decoys.

Database searches were performed using the parameters described in the above methods sections. The SEP-specific FDR was calculated by comparing true SEP detections to hits originating from the entrapment sequences. The randomly shuffled SEP database sequences resulted in higher SEP-specific FDRs then the marine archaeal databases and are therefore what are reported in this paper. The average SEP-specific FDR across samples analyzed on the Orbitrap Astral platform was 6.3% (±1.4%) (**Fig. 3A**). For comparison, identical samples analyzed on the Q Exactive Plus instrument using data-dependent acquisition (DDA) search results yielded an average small protein-specific FDR of 7.4% (±1.9%) (**Extended Data Fig. 5A**).

Supplemental Method 6:

Specifically, for peptide to protein roll-up, peptides mapping to one or more proteins within the same 95% sequence similarity and coverage cluster group were considered to be unique peptides and were included in abundance quantification for that cluster. Searching against 100% sequence similarity and coverage databases allowed for the capture of the biologically diverse protein sequence, while doing the peptide to protein roll-up at a 95% sequence similarity and coverage ensured that orthologous, functionally redundant, proteins were grouped together in clusters. Peptide to cluster roll-up was done separately for the SEP-specific identification to ensure that the only proteins being used were under 100 aa. Taxa (extrapolated from the databases)) were quantified by summing the intensities of all peptides that uniquely map to each taxon (If a peptide was mapped to multiple proteins, the lowest (most-specific) shared taxonomic rank among those proteins was identified. The peptide's abundance is then assigned to that common level and incorporated into the summed abundance for each of the corresponding taxonomic levels). A single peptide's intensity was added to all levels in the taxonomic hierarchy that the peptide could be uniquely attributed to. Taxon intensities were then divided by the sum of the total observed intensity of a sample to get the fraction of observed intensity attributable to a taxon. Functions were quantified by summing the intensities of all peptides that coherently mapped to each function. A coherent relationship is one in which all proteins that could generate a peptide are annotated with the function in question. This does not require that the peptide uniquely maps to a function. A single peptide can have a coherent relationship with multiple functions, even those of the same type, and its intensity would be added to all of those functions.
