# Supplementary material for: High-resolution multi-omics enhances prediction and detection of smORF-encoded proteins in the human gut microbiome
Source: Nat Commun. 2026 May 9;17:6296. doi: 10.1038/s41467-026-72762-5 (PMC13376575; doi:10.1038/s41467-026-72762-5)
Supplement: Supplementary file 2 — Description of Additional Supplementary Files [file 41467_2026_72762_MOESM2_ESM.pdf]

Description of Additional Supplementary Files

File Name: SupplementaryData1_BenchmarkingResults.csv

Description: Supplementary Data 1. A table with the smORF prediction workflow Benchmarking results.

File Name: SupplementaryData2_Unique_and_shared_predicted_smORFs_between_samples_Data.tsv

Description: Supplementary Data 2. A table with the unique and shared predicted smORFs between samples Data.

File Name: SupplementaryData3_Predicted_smORFs_sequence_length_distribution_Data.tsv

Description: Supplementary Data 3. A table with the predicted smORFs sequence length distribution data.

File Name: SupplementaryData4_Human-Microbial_Homology_Analysis_Results_At_Different_Identities_and_Coverages.xlsx

Description: Supplementary Data 4. A table with the human-microbial homology analysis results at different identities and coverages.

File Name: SupplementaryData5_ClusteringMetrics.csv

Description: Supplementary Data 5. A table with protein clustering metrics.

File Name: SupplementaryData6_Top_taxa_prevalence_in_each_taxonomic_rank.txt

Description: Supplementary Data 6. A table with the top taxa prevalence in each taxonomic rank for smORFs.

File Name: SupplementaryData7_Core_smORFs_taxonomic_annotation.tsv

Description: Supplementary Data 7. A table with the core smORFs taxonomic annotation

File Name: SupplementaryData8_Functional_annotation_of_core_smORFs.tsv

Description: Supplementary Data 8. A table with the functional annotation of core smORFs.

File Name: SupplementaryData9_DetectedProteins_95sc.xlsx

Description: Supplementary Data 9. A table showing detected proteins including SEPs for samples run on the Orbitrap Astral and Q Exactive Plus.

File Name: SupplementaryData10_SEP_Function_Taxonomy.xlsx

Description: Supplementary Data 10. A table with the SEP summed abundance to taxonomy/annotation.

File Name: SupplementaryData11_Global_Function_Taxonomy.xlsx

Description: Supplementary Data 11. A table with the protein global summed abundance to taxonomy/annotation.

File Name: SupplementaryData12_PerSampleContig_Statistics.tsv

Description: Supplementary Data 12. A table with the statistics for the per sample contig assemblies.
