# Supplementary material for: High-resolution multi-omics enhances prediction and detection of smORF-encoded proteins in the human gut microbiome
Source: Nat Commun. 2026 May 9;17:6296. doi: 10.1038/s41467-026-72762-5 (PMC13376575; doi:10.1038/s41467-026-72762-5)
Supplement: Supplementary file 15 — Reporting Summary [file 41467_2026_72762_MOESM15_ESM.pdf]

nature portfolio

Corresponding author(s): Paul Wilmes and Robert L Hettich

Last updated by author(s): Apr 17, 2026

Reporting Summary

Nature Portfolio wishes to improve the reproducibility of the work that we publish. This form provides structure for consistency and transparency in reporting. For further information on Nature Portfolio policies, see our Editorial Policies and the Editorial Policy Checklist.

Statistics

For all statistical analyses, confirm that the following items are present in the figure legend, table legend, main text, or Methods section.

n/a	Confirmed	
☐	☒	The exact sample size (*n*) for each experimental group/condition, given as a discrete number and unit of measurement
☐	☒	A statement on whether measurements were taken from distinct samples or whether the same sample was measured repeatedly
☒	☐	The statistical test(s) used AND whether they are one- or two-sided<br>*Only common tests should be described solely by name; describe more complex techniques in the Methods section.*
☒	☐	A description of all covariates tested
☐	☒	A description of any assumptions or corrections, such as tests of normality and adjustment for multiple comparisons
☐	☒	A full description of the statistical parameters including central tendency (e.g. means) or other basic estimates (e.g. regression coefficient) AND variation (e.g. standard deviation) or associated estimates of uncertainty (e.g. confidence intervals)
☒	☐	For null hypothesis testing, the test statistic (e.g. *F*, *t*, *r*) with confidence intervals, effect sizes, degrees of freedom and *P* value noted<br>*Give P values as exact values whenever suitable.*
☒	☐	For Bayesian analysis, information on the choice of priors and Markov chain Monte Carlo settings
☒	☐	For hierarchical and complex designs, identification of the appropriate level for tests and full reporting of outcomes
☒	☐	Estimates of effect sizes (e.g. Cohen's *d*, Pearson's *r*), indicating how they were calculated

Our web collection on statistics for biologists contains articles on many of the points above.

Software and code

Policy information about availability of computer code

Data collection	Multi-omics data were generated using the following acquisition software: XCalibur V4.7 (Vanquish Neo UHPLC + Orbitrap Astral, DIA), XCalibur V4.0 (Vanquish UHPLC + Q Exactive Plus, DDA); raw files processed in Proteome Discoverer V3.2 (Thermo Scientific) with Chimera. Data processing used IMP V202402_v02 (metagenomics/metatranscriptomics), pyrodigal-gv V0.3.2 (smORF prediction), MMseqs2 V15.6f452 (clustering/taxonomy), Mantis V1.5.5 (annotation), plus Infernal V1.1.4, Aragorn V1.2.41.c, AntiFam V8.0 (filtering). Custom pipelines for smORF prediction/database construction implemented in Python/Bash/Snakemake; full code/parameters in the Methods section. Sequencing: Dragon BioIT V4.2.7.
Data analysis	Metagenomic/metatranscriptomic data was processed using the latest version of IMP (version 202402_v02) and smORF predictions were obtained using the custom smORF prediction pipeline, which is available on gitlab (https://gitlab.com/uniluxembourg/lcsb/systems-ecology/smorf_methods_paper) and described on the paper. Metaproteomic run files analyzed with Proteome Discoverer V3.2.0, R V4.5.1, and Python V3.13

For manuscripts utilizing custom algorithms or software that are central to the research but not yet described in published literature, software must be made available to editors and reviewers. We strongly encourage code deposition in a community repository (e.g. GitHub). See the Nature Portfolio guidelines for submitting code & software for further information.

Data

Policy information about availability of data

All manuscripts must include a data availability statement. This statement should provide the following information, where applicable:

- Accession codes, unique identifiers, or web links for publicly available datasets
- A description of any restrictions on data availability
- For clinical datasets or third party data, please ensure that the statement adheres to our policy

Metagenomic and metatranscriptomic files are available through European Read Archive ENA under Project ID PRJEB97797. All proteomic raw files, and corresponding search results are deposited in the Proteome XChange Consortium via the MassIVE repository under the ID MSV000099334 [doi:10.25345/C50V89W6F]. Source data is available within the listed repositories and within this paper.

Research involving human participants, their data, or biological material

Policy information about studies with human participants or human data. See also policy information about sex, gender (identity/presentation), and sexual orientation and race, ethnicity and racism.

Reporting on sex and gender	Not applicable to the study.
Reporting on race, ethnicity, or other socially relevant groupings	Not applicable to the study.
Population characteristics	Not applicable to the study.
Recruitment	Participants were recruited at two specialised clinical sites in Germany (Charité—Universitätsmedizin Berlin and Paracelsus-Elena Clinic in Kassel) via physician referrals from affiliated outpatient departments or non-personal advertising (e.g., flyers, social media) as outlined in https://bmjopen.bmj.com/content/13/8/e071380#boxed-text-2. For this analysis, only baseline cross-sectional data from the cohort of interest were included. Eligible individuals provided written informed consent and were assigned to their respective groups after screening against prespecified inclusion/exclusion criteria.
Ethics oversight	Ethical approval was obtained to plan and conduct the trial from the institutional review board of the Charité-Universitätsmedizin Berlin (EA1/204/19), the ethics committee of the state medical association (Landesärztekammer) of Hessen (2021-2230-zvBO) and the Ethics Review Panel (ERP) of the University of Luxembourg (ERP 21-001 A ExpoBiome).

Note that full information on the approval of the study protocol must also be provided in the manuscript.

Field-specific reporting

Please select the one below that is the best fit for your research. If you are not sure, read the appropriate sections before making your selection.

☒ Life sciences ☐ Behavioural & social sciences ☐ Ecological, evolutionary & environmental sciences

For a reference copy of the document with all sections, see nature.com/documents/nr-reporting-summary-flat.pdf

Life sciences study design

All studies must disclose on these points even when the disclosure is negative.

Sample size	Sample size calculations do not apply here because no statistical analysis were performed. The sample size is 41 healthy volunteers.
Data exclusions	The full ExpoBiome study includes a total of 60 healthy volunteers, of which 41 were studied here. These 41 samples had sufficient sample material to perform paired analysis on the Q Exactive and Orbitrap Astral mass spectrometers.
Replication	No replicates were performed, however, paired experiments from the same starting material were performed on two different mass spectrometers. The extent of the overlaps are described in the manuscript.
Randomization	Randomized batches were used in the DNA, RNA and protein extractions.
Blinding	No blinding.

Reporting for specific materials, systems and methods

We require information from authors about some types of materials, experimental systems and methods used in many studies. Here, indicate whether each material, system or method listed is relevant to your study. If you are not sure if a list item applies to your research, read the appropriate section before selecting a response.

Materials & experimental systems

n/a	Involved in the study
☒	☐ Antibodies
☒	☐ Eukaryotic cell lines
☒	☐ Palaeontology and archaeology
☒	☐ Animals and other organisms
☐	☒ Clinical data
☒	☐ Dual use research of concern
☒	☐ Plants

Methods

n/a	Involved in the study
☒	☐ ChIP-seq
☒	☐ Flow cytometry
☒	☐ MRI-based neuroimaging

Clinical data

Policy information about clinical studies
All manuscripts should comply with the ICMJE guidelines for publication of clinical research and a completed CONSORT checklist must be included with all submissions.

Clinical trial registration	NCT04847011
Study protocol	https://bmjopen.bmj.com/content/13/8/e071380
Data collection	For this analysis, we used only baseline, cross-sectional data from ExpoBiome, focusing on the cohort of interest. At the baseline visit (T0), participants provided blood, stool, saliva and midstream urine samples, and completed standardized questionnaires on dietary behaviour, lifestyle, general health and well-being. Biospecimens and questionnaires were collected on the same day at the clinical study sites according to the prespecified protocol and were recorded in pseudonymised electronic case report forms.
Outcomes	Primary outcome: molecular profiling of the gut microbiota using stool based sequencing and high throughput analyses (metagenomics, metatranscriptomics, metaproteomics, metabolomics). Secondary outcomes of the overall clinical study are not applicable to this manuscript submission.

Plants

Seed stocks	Not applicable.
Novel plant genotypes	Not applicable.
Authentication	Not applicable.
