# Supplementary material for: High-resolution multi-omics enhances prediction and detection of smORF-encoded proteins in the human gut microbiome
Source: Nat Commun. 2026 May 9;17:6296. doi: 10.1038/s41467-026-72762-5 (PMC13376575; doi:10.1038/s41467-026-72762-5)
Supplement: Supplementary file 16 — Transparent Peer Review file [file 41467_2026_72762_MOESM16_ESM.pdf]

nature portfolio

Peer Review File

High-resolution multi-omics enhances prediction and detection of smORF-encoded proteins in the human gut microbiome

Corresponding Author: Professor Paul Wilmes

This file contains all reviewer reports in order by version, followed by all author rebuttals in order by version.

Version 0:

Reviewer comments:

Reviewer #1

(Remarks to the Author)
The manuscript entitled “Expanding the human metaproteome: enhancing small open reading frame (smORF) prediction and ultra-deep detection of smORF-encoded proteins in the gut microbiome” presents an investigation of smORF-encoded proteins from gut microbiomes. The authors apply different proteomics protocols and introduce a computational approach to identify and quantify smORF-encoded proteins in microbiomes. They subsequently perform taxonomic and functional analyses on the results.

General Comments:
While the manuscript is generally well written, the soundness of the approach for identifying smORF-encoded proteins is not evident. There are issues with FDR estimation. The authors also claim their method is novel, which may very well be the case, but they provide no benchmark against currently applied or previously proposed methods. In addition, important methodological information is missing, preventing readers from replicating the approach and thereby severely limiting the impact of the work.

Major Comments:
Due to missing methodological details, the computational pipeline would be extremely difficult to reproduce. What parameters were used for software such as AntiFam, Infernal, and Aragorn? With so little information, it is virtually impossible to assess the soundness of the methods. Regarding the database search, did the authors search for fully tryptic or half-tryptic peptides? What was the mass tolerance?
The methods indicate that the authors allow searching for peptides as short as 7 aa, but line 275 states that “our prediction pipeline included smORFs as short as 8 aa.” Which is correct?

The human smORF database used is far too small. Many more human smORFs have been reported in resources such as OpenProt. Using such a limited human smORF database may lead to shared peptides between human and bacterial smORFs to be completely missed, potentially confounding the results.

The finding that only 0.6% of SEPs originate from humans is difficult to believe. In stool samples, the overwhelming majority of proteins are human-derived. This strongly suggests that the human smORF database used is incomplete and many of them are missed. An excessively bacteria-dominated database increases the likelihood of random spectral matches to bacterial smORFs.

It is also unclear whether the entrapment database used here is appropriate. Marine archaea peptides likely have different amino acid distributions than those from gut microbes. Accurate FDR estimation assumes that random matches are equally likely to occur in the entrapment database and the actual database. If the entrapment database does not reflect the characteristics of the real data, this assumption is violated, resulting in unreliable and optimistic FDR estimates. Given that the reported FDRs are already high, this is a significant concern. Moreover, the entrapment database does not represent human proteins, which constitute a large fraction of the sample.

The authors claim to “present a customized novel pipeline for high-quality, comprehensive smORF prediction,” but they do

not benchmark their method against any currently used or previously proposed approaches. Benchmarking is essential to contextualize the work and assess its impact.

When the authors state that smORFs are unique to a sample, do they mean the exact same amino acid sequence not being present in any other samples? If so, this is not particularly informative: one would expect a number of smORFs from different species across samples to show high level of similarity, while being conserved.

To assess the recurrence of SEPs across samples, it would have been important to compare this to the recurrence of non-SEPs. It would also have been a valuable control to compare, within a given species, the intensities of SEPs and non-SEPs.

Minor Comments:
The use of the term “next-generation MS platforms” is unclear. If this refers to a Thermo TOF instrument (Astral) using DIA, then it should be described explicitly. While these instruments are faster and enable rapid DIA scanning, the underlying concepts are not really new per se. In addition, it is not clear how such a “next-generation MS platform” eliminates the need for de novo peptide sequencing. This improvement is more attributable to advances in computational metaproteomics and should be referenced accordingly.

Line 577: The meaning of the filter “completeness (i.e., partial = 00)” is unclear.

Line 365: Typographical error: missing space in “SEPsdetected”

(Remarks on code availability)
The documentation is minimal and not sufficient to reproduce the results.

Reviewer #2

(Remarks to the Author)
Davin et al. present a careful study of the metaproteome of the human gut microbiome. The research is very interesting and the manuscript is well written. The field certainly needs improved workflows and standardization of protocols, and I commend the authors on their work on these. Two aspects that are quite novel are firstly use of the Orbitrap Astral technology which seems to allow detection of many more proteins. Secondly, including statistics on abundance is very interesting and quite novel. Perhaps the novel aspects could be highlighted more clearly e.g. in the abstract, and perhaps the increase over previous studies could be noted.

Substantive points

There is very little in the methods section about the underlying genomes (contigs) used to build the database, usually referred to as “metagenome assembled genomes” (MAGs) - please clarify the number of genomes or contigs present and their quality statistics. Similarly, there is almost nothing in the methods regarding taxonomic assignment. What does it mean to “map to a taxon” here? Is this based on the contigs, or on EGGNOG, or something else?

A comparison to previous catalogues such as the GMSC (how much overlap is there?) would be relevant, if there is space for this.

This sentence “The detected taxonomic species account for less than 10% seen in the metagenomic data” is a key point which should be briefly explained. Is this due partly to the methods used for taxonomic assignment of smORFs, and/or perhaps low abundance of some species?

The requirement for a ribosome binding site deserves brief discussion. What is the proportion of small proteins expected to have a RBS site?

Minor points

Fig 3D - I suggest (at your discretion) using a different visualisation to allow including category “S” - e.g. bar plots on log scale. It is unclear why unclassified orders needed to be excluded, they could be an additional row.

It is stated that “Overall, the taxonomic make-up of these samples is relatively consistent with the global proteome taxonomic profiles, validating that we are not observing spurious small protein hits but rather biologically meaningful patterns” but it is not clear how this follows. Spurious proteins would also follow the taxonomic distribution of the underlying genomes - please clarify.

Line 333: “does not fit within” - typo.

(Remarks on code availability)
I have only briefly reviewed the code and note that for the bash script to run the snakemake workflow, the directories appear to be hard coded, they should probably be passed as arguments instead if it is to be usable by others.

Version 1:

Reviewer comments:

Reviewer #1

(Remarks to the Author)
The authors have addressed some of my concerns, but several important ones remain.

1. It seems questionable to search for fully tryptic peptides given the small size of ORFs. One would expect many peptides to be semi-tryptic or not tryptic at all due to the very few opportunities for trypsin digestion.

2. The benchmark proposed is highly problematic. While the methods proposed may be more sensitive, a tool that calls everything an ORF would also appear more sensitive. The error rate (or detection rate) of predictions from smORFinder is vastly better: 16 out of 17 compared to the authors' tool (42 out of 450). The authors also do not provide any FDR assessment for the results from either tool. Protein identification numbers cannot be meaningfully compared without reporting FDRs. The authors should have at least provided performance curves (number of identifications) for both tools at different and comparable FDR thresholds.

At this stage, I remain far from convinced that the proposed tool represents an improvement in validation compared to the other tool, contrary to the authors' claims. Furthermore, the benchmarking remains limited, as only a single tool is tested across two datasets.

3. The FDRs reported in the study remain high and are in fact even higher now that the entrapment strategy is used and seem to be more representative of the data. The authors claim that a 5% FDR threshold is widely accepted and cite a single study from Proteomics. This claim is greatly exaggerated. This threshold is not widely accepted. While users can choose the FDR threshold they are willing to accept in their experiments, given the concerns regarding specificity, the authors should report all of their results at a 1% FDR.

(Remarks on code availability)
The authors have improved the documentation.

Reviewer #2

(Remarks to the Author)
My previous review comments have been addressed well - well done to the authors on the careful responses to reviewer comments, and the detailed work in this study.

I note just two minor points:

The sentence "high-resolution taxonomic assignments are challenging in metaproteomics due to genomes within more-specific taxonomic ranks (i.e. family, or species) having high homology)" should be corrected to something like "high similarity" as homology, technically, does not differ by degrees.

Line 213: typo - coverages

(Remarks on code availability)

Open Access This Peer Review File is licensed under a Creative Commons Attribution 4.0 International License, which permits use, sharing, adaptation, distribution and reproduction in any medium or format, as long as you give appropriate credit to the original author(s) and the source, provide a link to the Creative Commons license, and indicate if changes were made.
In cases where reviewers are anonymous, credit should be given to 'Anonymous Referee' and the source.
The images or other third party material in this Peer Review File are included in the article's Creative Commons license, unless indicated otherwise in a credit line to the material. If material is not included in the article's Creative Commons license and your intended use is not permitted by statutory regulation or exceeds the permitted use, you will need to obtain permission directly from the copyright holder.
To view a copy of this license, visit https://creativecommons.org/licenses/by/4.0/

REVIEWER COMMENTS: Our Responses

We would like to thank all the reviewers for their thorough review of the manuscript. We appreciate their comments and believe that these contributions have significantly improved the paper. Below are our responses to each comment along with the corresponding manuscript text for readability. Within the materials submitted with our reviewer response, in addition to the clean updated manuscript, we have included an updated manuscript version that highlights our edits and additions to easily identify the changes made.

Reviewer #1 (Remarks to the Author):

The manuscript entitled "Expanding the human metaproteome: enhancing small open reading frame (smORF) prediction and ultra-deep detection of smORF-encoded proteins in the gut microbiome" presents an investigation of smORF-encoded proteins from gut microbiomes. The authors apply different proteomics protocols and introduce a computational approach to identify and quantify smORF-encoded proteins in microbiomes. They subsequently perform taxonomic and functional analyses on the results.

General Comments:

1. While the manuscript is generally well written, the soundness of the approach for identifying smORF-encoded proteins is not evident. There are issues with FDR estimation. The authors also claim their method is novel, which may very well be the case, but they provide no benchmark against currently applied or previously proposed methods. In addition, important methodological information is missing, preventing readers from replicating the approach and thereby severely limiting the impact of the work.

We thank you for your assessment of our manuscript and work. In order to address the concerns, we have benchmarked our prediction pipeline against smORFinder (see **comment 7**), and have run a separate FDR estimation with an entrapment database that comes from a random selection of shuffled sequences from the real smORF databases (including human) (see **comment 6**); each of these actions is detailed in the text below. We have also revised the respective methods sections to include necessary and relevant information to ensure replicability of our work (see **comment 2**).

Major Comments:

2. Due to missing methodological details, the computational pipeline would be extremely difficult to reproduce. What parameters were used for software such as AntiFam, Infernal, and Aragorn? With so little information, it is virtually impossible to assess the soundness of the methods. Regarding the database search, did the authors search for fully tryptic or half-tryptic peptides? What was the mass tolerance?

Thank you for pointing this out; we realize that we did not include several of the parameters needed to reproduce the pipeline. This has now been amended. In the section "smORF prediction pipeline" (starting on line 665) and the section "Metaproteomics Database Construction" (starting on line 712). In these sections, we describe the parameters used for AntiFam, Infernal and Aragorn including in lines 683-698:

> "To eliminate spurious ORF predictions, the pipeline incorporates three filtering tools. AntiFam (version 8.0)[58] was applied using pyhmmer with gathering threshold bit score cutoffs to identify spurious protein-coding sequences based on curated hidden Markov models. Infernal (version 1.1.4; RRID:SCR_011809)[59] was run with cmscan using parameters -rfam --fmt 2 --nohmmonly --cut_tc -g –noali to detect non-coding RNAs against the Rfam database. Aragorn (version 1.2.41.c; RRID:SCR_015974)[60], was executed twice with parameters –d –w –fasta, using both standard (-gcstd) and bacterial (-gcbact) genetic codes to identify tRNA and tmRNA genes. Predicted smORFs matching any of these databases were excluded from the final per-sample smORF dataset used for the metaproteomics database construction. AntiFam is a curated database of hidden Markov models that are specifically designed to identify spurious protein-coding sequences in genome and metagenome datasets[58]. Infernal is used for searching, aligning, and annotating RNA sequences based on both sequence and secondary structure. It can be used to detect misannotated non-coding RNAs mistakenly predicted as proteins, therefore, being useful to remove false positive entries from the dataset[59]. Aragorn is a tool designed to detect tRNA and tmRNA genes in nucleotide sequences and, on the same line, it can help to remove spurious protein predictions from a database[60]."

In addition, we have also included the MMseqs2 parameters for dereplication and for the taxonomic annotations in the "Metaproteomics database construction section" (starting on line 705), including the following in lines 718-721:

> "We then downloaded the human reference proteome from NCBI (version 2025-06-10; GRCh38.p14; RRID:SCR_006472)[27], removed duplicate entries with MMseqs2 easy-cluster with the parameters -c 1.0 --min-seq-id 1.0 --cov-mode 0 --cluster-mode 0 (version 15.6f452; RRID:SCR_022962)…"

And in lines 725-729:

> "We concatenated the IMP and smORF protein datasets for each sample and removed redundant sequences using MMseqs2 easy-cluster with the parameters -c 1.0 --min-seq-id

1.0 --cov-mode 0 --cluster-mode 0 (version 15.6f452; RRID:SCR_022962). In parallel, we merged the human and cRAP database and eliminated duplicates using the same MMseqs2 parameters (version 15.6f452; RRID:SCR_022962)."

Moreover, we have added the following to the proteomics methods in lines 772-774:

"Both searched for fully-tryptic peptides with a 7 aa minimum peptide length, 2 ppm mass tolerance, 2 max missed cleavages, and 1% peptide level FDR."

3. The methods indicate that the authors allow searching for peptides as short as 7 aa, but line 275 states that "our prediction pipeline included smORFs as short as 8 aa." Which is correct?

We appreciate you pointing out this discrepancy. This was a typo and has been corrected to "7 aa" on line 324 (formerly line 275).

4. The human smORF database used is far too small. Many more human smORFs have been reported in resources such as OpenProt. Using such a limited human smORF database may lead to shared peptides between human and bacterial smORFs to be completely missed, potentially confounding the results.

Thank you for this comment. We see why this is a concern and to address it, we expanded the human smORF database to include a comprehensive catalogue from three sources: the human reference proteome, the 7,554 human smORFs from Martinez et al. (https://doi.org/10.1038/s41589-019-0425-0), and 150,882 OpenProt human smORFs supported by at least two unique peptides (https://academic.oup.com/nar/article/52/D1/D522/7416803). We then assessed potential human–microbial smORF homology by clustering the combined human and microbial smORFs with MMseqs2 at multiple identity/coverage thresholds. No mixed human–microbial clusters were detected at stringent thresholds (100% identity / 100% coverage and 95% identity / 95% coverage, respectively), and only 4 human smORFs clustered with microbial smORFs at a more relaxed threshold (90% identity 80% coverage). These few cases correspond to highly conserved protein families (Ras/RAB2B-associated, histone H3 variants, ubiquitin-like), indicating that even with OpenProt included, shared human-microbial smORFs are extremely unlikely to confound our peptide assignments. These results have now been included in the manuscript in lines 204-215:

"To confirm minimal human-microbial smORF homology, that could result in the misassignment of spectra to the wrong peptides, we clustered a comprehensive human smORF catalogue, (reference proteome smORFs[27] + 7,554 from Martinez et al.[29] + 150,802 from OpenProt[30]; clustered via MMseqs2[26] at multiple identity/coverage thresholds) with microbial smORFs. There were no mixed clusters at stringent thresholds (100% identity / 100% coverage and 95% identity / 95% coverage) and only 4 at relaxed thresholds (90%

identity / 80% coverage; **Supplemental Table 4**), mapping to conserved families (Ras/RAB2B, histone H3 variants, ubiquitin-like). The latter thresholds were more relaxed than the parameters typically used in microbiome smORF studies, which commonly use identities and covarges between 90% and 95%[7,8]. With minimal overlap confirmed, subsequent analyses focused on microbial smORFs/SEPs. Full catalogue details, results, and parameters in **Supplemental Note 2**."

We also include a new section in the methods to explain the approach we used in lines 741-746:

"A human smORF catalogue (1,509 from reference proteome[27] + 7,554 from Martinez et al.[29] + 150,802 from OpenProt[30]; final dereplicated: 157,901) was concatenated with microbial smORFs from our database and clustered via MMseqs2 easy-cluster at different thresholds[26]. Mixed clusters (≥1 human + 1 microbial smORF) were identified; human annotations were obtained from OpenProt[30]. Full catalogue construction, parameters and results are found in **Supplemental Method 2**."

as well as in the discussion in lines 517-522:

"Expanding the human smORF catalogue for metaproteomics to 157,901 dereplicated sequences yielded few smORFs shared between microorganisms and humans. Using loose matching criteria homologous sequences were restricted to core families like histones, ubiquitin-like systems, and Ras GTPases[41-43]. This rarity of cross-kingdom homology indicates lineage-specific evolution and negligible misassignment risk from using only 1,509 reference human smORFs, as shared sequence space is extremely limited. Full details can be found in **Supplemental Discussion 2**."

5. The finding that only 0.6% of SEPs originate from humans is difficult to believe. In stool samples, the overwhelming majority of proteins are human-derived. This strongly suggests that the human smORF database used is incomplete and many of them are missed. An excessively bacteria-dominated database increases the likelihood of random spectral matches to bacterial smORFs.

For fecal, gut-microbiome samples, the average human protein contribution can vary greatly by sample type but in many cases the abundance is around 10% (https://www.nature.com/articles/nmicrobiol2016180). Our global proteome does show that our average protein abundance for human contribution in these samples is around 6.4%. The literature, to our knowledge, does not have any detected protein reports of the percent of human SEPs within a fecal microbiome sample. Additionally, the focus of this paper is to increase identification of microbial SEPs within samples from the human gut microbiome and, while interesting, it does not focus on the identification of human SEPs.

To address the concern of random spectral matches to microbial SEPs, we performed a human-microbial smORF homology analysis, as explained in **comment 4**.

6. It is also unclear whether the entrapment database used here is appropriate. Marine archaea peptides likely have different amino acid distributions than those from gut microbes. Accurate FDR estimation assumes that random matches are equally likely to occur in the entrapment database and the actual database. If the entrapment database does not reflect the characteristics of the real data, this assumption is violated, resulting in unreliable and optimistic FDR estimates. Given that the reported FDRs are already high, this is a significant concern. Moreover, the entrapment database does not represent human proteins, which constitute a large fraction of the sample.

Your point is fair and well-taken. To address this concern and in addition to our previous analyses, we took all the smORFs (both microbial and human) from our databases and randomly shuffled their amino acid sequences. From this database, entrapment sequences were randomly selected and added to the smORF databases at a 1:1 (real database smORF to entrapment smORF) ratio. This was done with the 12 randomly selected samples previously selected, and the FDRs were then re-calculated. This should address the relevance of the entrapment database concern by matching the amino acid characteristics to the actual database. The methods have been updated to reflect this new FDR analysis in lines 777-785(and further in **Supplemental Method 5**):

> "To evaluate the accuracy of the SEP detection, a SEP-specific false discovery rate (FDR) at the peptide level calculations was calculated first with an entrapment database comprised of marine archaea sequences[32], then secondly, an entrapment database created from compiling the SEP database sequences and randomly shuffling each sequence. The creation of these databases and the calculations are further detailed in **Supplemental Method 5**. These SEP-specific FDR calculations were done for 12 randomly selected samples, for both the Orbitrap Astral platform (**Fig. 3A**), and the Q Exactive Plus instrument runs (**Extended Data Fig. 5A**). While the SEP-specific FDR exceeded the conventional 1% threshold, it remained near the widely accepted more liberal 5% peptide FDR cutoff commonly used in proteomic studies[44]."

These re-calculations show that for the Q Exactive, the new average FDR (7.4%) slightly rose compared to the previous FDR (6.0%). For the Astral, the average FDR rose slightly as well to 6.3% from 4.9%. These new FDRs increased just above 1% from the original SEP-specific FDR calculations. These new percentages are now represented in **Fig. 4A** and in the text for the Orbitrap Astral in lines 312-314:

> "The detected SEPs in these samples maintained a SEP-specific (peptide level) FDR (achieved with a shuffled entrapment database approach) across samples at a mean FDR

of 6.3% (±1.4%), validating that the large number of SEPs detected reflect confident identifications (**Fig. 4A**)."

and for the Q Exactive FDR calculations in **Extended Fig. 5** as well as in lines 338-340:

"The same samples analyzed with the Astral Orbitrap were run with the Q Exactive Plus. The results of these runs yielded a slightly higher SEP-specific (peptide level) FDR of 7.4% (±1.9%)…"

7. The authors claim to "present a customized novel pipeline for high-quality, comprehensive smORF prediction," but they do not benchmark their method against any currently used or previously proposed approaches. Benchmarking is essential to contextualize the work and assess its impact.

Your point is fair and well-taken. To address this concern, we added a benchmarking analysis to contextualize the performance of our smORF prediction pipeline against the state-of-the-art method, smORFinder, using *E. coli* K-12 substrain *MG1655*, which has 536 EcoCyc-validated small proteins (164 of which are less than 50 amino acids). We used the EcoCyc database as it is a well-curated database that includes experimentally validated small proteins from *E. coli* K-12 substrain MG1655. To our knowledge, there is no database for metagenomic data that includes both the regular genes and the small open reading frames that have been validated experimentally to use for benchmarking between the two tools. In addition, we also ran smORFinder on our co-assembled contigs to directly compare predictions on our metagenomic/metatranscriptomic dataset. SmORFinder predicted 77,027 non-redundant smORFs of ≤50 amino acids, whereas our pipeline predicted 524,743 smORFs. Of the smORFs predicted by smORFinder, 76,261 (99%) were also predicted by our pipeline, demonstrating that our method captures nearly all smORFinder predictions while identifying substantially more candidates. We then evaluated proteomic support for the smORFinder predictions: 628 smORFs were detected in our metaproteomics-validated dataset, which contains 1921 SEPs of ≤50 amino acids. Finally, we mapped smORFs from both approaches to the GMSC to assess their representation in a large-scale reference database. Of the smORFs predicted by our pipeline, 448,530 (85.47%) had homologs in GMSC, compared to 71,542 (92.88%) of smORFinder-predicted smORFs. These results demonstrate that our pipeline provides comprehensive smORF prediction with high overlap with existing methods while substantially expanding the predicted smORF repertoire, the majority of which have support from an independent global catalogue. The results of these analyses have been included in the manuscript in lines 137-149:

"We benchmarked our pipeline against smORFinder using *E. coli* K-12 substrain MG1655 (536 EcoCyc-validated small proteins ≤100 aa, 164 ≤50 aa)[5,25]. SmORFinder predicted 17 smORFs ≤50 aa, while our prediction pipeline predicted 450 (91 ≤50 aa) (**Supplemental Table 1**). MMseqs2-based validation using EcoCyc showed that smORFinder correctly

predicted 16/164 (9.8%) while our prediction pipeline predicted 42/164 (25.6%) ≤50 aa and 342/536 (63.8%) ≤100 aa (**Supplemental Table 1**)[26]. On the co-assembled contigs, smORFinder predicted 77,027 non-redundant smORFs ≤50 aa, with 628 having metaproteomic detection. Our prediction pipeline predicted 524,743 smORFs, with 76,261 also being predicted by smORFinder and 1,912 having been identified by metaproteomics. The full results and parameters for this section are found in **Supplemental Note 1**. Overall, our approach substantially improved small protein recovery and validation rates, yielding markedly higher sensitivity against EcoCyc-validated proteins and identifying a greater number of metaproteomics-supported smORFs compared to smORFinder."

In addition, we also included a methods section to describe the procedure in lines 704- 711:

"We benchmarked our smORF prediction pipeline against smORFinder[5] using *Escherichia coli* K-12 substrain MG1655 (NC_000913.3), which EcoCyc reports as having 536 validated small proteins ≤100 aa (164 ≤ 50aa)[25]. Predictions from both tools were validated against EcoCyc proteins using MMseqs2 easy-search at 100% identity and 90% coverage to account for boundary differences[26]. We also ran our study's co-assembled contigs on smORFinder and compared the results to ours using MMSeqs2 (100% identity 90% coverage)[26]. GMSC-mapper was run on both datasets to obtain homologs to a published smORF catalogue[8,32]. Full benchmarking details, parameters, and results are in **Supplemental Method 1**."

as well as a section in the discussion in lines 511-516:

"Although developed for gut metagenomic assemblies rather than isolate genomes, our pipeline outperformed smORFinder on *E. coli* K-12 MG1655 under both stringent and relaxed criteria, with consistent boundary prediction and expected short-gene limited recovery[40]. On the co-assembled contigs, most smORFinder predictions overlapped with ours, though only a subset showed metaproteomic support. Benchmarking details and full discussion are available in **Supplemental Discussion 1**."

8. When the authors state that smORFs are unique to a sample, do they mean the exact same amino acid sequence not being present in any other samples? If so, this is not particularly informative: one would expect a number of smORFs from different species across samples to show high level of similarity, while being conserved.

In the predicted smORFs section, we present the smORFs as they are found in the metaproteomics databases used for database searching. In order to capture sequence variability and enable sensitive peptide database matching, the sequences were initially only dereplicated and raw proteome files were searched against 100% identity and 100% coverage sample-specific databases to capture biological variability. At the reviewer's suggestion, and to address concerns regarding protein-level interpretation, we additionally applied 95% sequence identity and 95% coverage clustering

for the downstream protein-level analysis. This clustering threshold groups highly homologous SEPs while reducing redundancy and allowing for more accurate protein counts across samples. The selected sequence identity and coverage threshold was decided based on prior smORF studies (https://pubmed.ncbi.nlm.nih.gov/33290720/). The new sections detailing the identity/coverage clustering are described in the results in lines 217-224:

> "We clustered the metaproteomics database at 95% identity and 95% coverage, as used previously in other studies[5]. The clustering yielded 3,060,759 representative smORFs, representing an 18.6% reduction from the original database (**Supplemental Table 5**). Of these clusters, 86.5% were singletons, while 13.5% formed multi-member clusters with a mean size of 1.23 (**Supplemental Table 5**). Clustered smORFs showed increased sample prevalence, with 22.2% of the smORFs being found in two samples or more (**Supplemental Table 5**). This approach reduces complexity while preserving biological diversity, thereby allowing the grouping of functionally similar protein variants together for metaproteomic biological interpretation."

and in the methods in lines 750-752:

> "The metaproteomic database was clustered with MMseqs2 easy-cluster at 95% identity and 95% coverage as used in previous microbial smORF studies to obtain biological interpretation[26]. Full workflow in **Supplemental Method 4**."

Furthermore, the use of these databases was detailed again within the metaproteomics results sections in lines 301-305:

> "To capture biological variability, metaproteomes were searched against 100% identity and 100% coverage sample-specific databases. Then, as detailed above, peptides were clustered at 95% sequence and coverage similarity for protein-level analysis. This clustering threshold groups highly homologous SEPs allowing for accurate protein counts across samples."

All proteomic protein figures and summary statistics in the manuscript were updated to reflect the 95% sequence identity and coverage clustering. Updates were made throughout the manuscript, including throughout the section, "Detection of SEPs within the metaproteome", starting at line 299 with the most notable edits made in lines 314-324:

> "An average of just over 1,600 SEPs were detected per sample (representing 7.6% of the total proteome protein count), with a cumulative total of 25,233 unique SEPs across the sample set (**Fig. 4B & C**). These numbers exceeded previous SEP detections reported in the human gut microbiome and elsewhere, either from studies that employed size-based enrichment of small proteins or those that measured the entire proteome[19,20]. Additionally, SEPs accounted for up to 6.3% of the untargeted proteome abundances in individual samples, with a mean contribution of 3.6% (±0.9% standard deviation). The majority of these SEPs clustered within the 85–100 aa range, representing 40% of all detected SEPs.

In contrast, only 13 SEPs below 25 aa in length were detected, with the smallest detected SEPs measuring at just 21 aa, even though our prediction pipeline included smORFs as short as 7 aa (**Fig. 2B & 4D**).”

At the per-sample level, the average number of detected SEPs increased by approximately 100 proteins, to ~1,608 SEPs per sample (from ~1,573), while the total number of unique SEPs across the dataset decreased to 25,233 (from ~30,163). This increase in per-sample protein counts is expected, as peptides that were previously shared among highly similar sequences are now consolidated within 95% sequence clusters and contribute to a single representative protein. Conversely, the reduction in the total number of detected proteins reflects the collapse of highly homologous SEPs into shared clusters, resulting in more conservative protein-level estimates and an increased representation of conserved proteins across samples.

9. To assess the recurrence of SEPs across samples, it would have been important to compare this to the recurrence of non-SEPs. It would also have been a valuable control to compare, within a given species, the intensities of SEPs and non-SEPs.

We agree that comparing SEP recurrence to that of non-SEPs, as well as evaluating recurrence within a given species, are important controls and have now incorporated these analyses into the manuscript. First, to address overall recurrence patterns, we added a supplemental figure comparing SEP and non-SEP recurrence across samples (**Supplemental Fig. 2**) and added comparison metrics in lines 349-351:

“Within all samples, only 605 SEPs (2.3% of all detected SEPs) appeared in 20 or more of the samples (**Fig. 4C**). This number of conserved SEPs is comparable to what is seen in the global data whereby only 3.2% of proteins are detected in 20 or more samples (**Supplemental Fig. 2B**).”

Second, to assess recurrence within a specific taxonomic group, we evaluated SEPs and non-SEPs from Clostridia (order Lachnospirales), selected based on their high abundance in the dataset (**Fig. 5A**). These results are presented in **Supplemental Fig. 3** and discussed in lines 353-359:

“When looking at conservation within Clostridia Lachnospirales, the taxonomic order with the highest SEP abundance (**Fig. 5A**), SEPs identified in 20 or more samples represent 3.2% of the total identified Lachnospirales SEPs, similar to the percentage of total Lachnospirales proteins (including proteins above 100 aa) identified in 20 or more samples, which was 5.0%. This shows the slightly more conserved nature of Lachnospirales proteins compared to the total SEP and global proteome profiles (**Supplemental Fig. 3**).”

Minor Comments:

10. The use of the term "next-generation MS platforms" is unclear. If this refers to a Thermo TOF instrument (Astral) using DIA, then it should be described explicitly. While these instruments are faster and enable rapid DIA scanning, the underlying concepts are not really new per se. In addition, it is not clear how such a "next-generation MS platform" eliminates the need for de novo peptide sequencing. This improvement is more attributable to advances in computational metaproteomics and should be referenced accordingly.

We agree that this wording is unclear, and thus we have changed this wording to specify that we are using the Astral instrument in line 104-105:

> "New instrumentation, such as the Orbitrap Astral, offers greatly improved measurement metrics in speed, throughput, and sensitivity[23]."

And in line 111-114:

> "These databases are then mined with mass spectral data from the newest generation of high-performance MS instrumentation, the Orbitrap Astral, for ultra-deep untargeted metaproteome characterizations, including both large, annotated proteins as well as SEPs within the same measurement."

These new generation, high-scanning speed MS instrumentation platforms dramatically expand the depth and range of quality mass spectral data, and thus, when combined with our tool to more comprehensively include smORFs in the metagenomes, enable a much deeper detection and characterization of SEPs which are largely excluded from normal search databases. The method doesn't eliminate the need for de novo, but rather offers an alternative to detecting them, as de novo sequencing comes with its own challenges and often high false discovery rate when looking at small peptides and proteins.

11. Line 577: The meaning of the filter "completeness (i.e., partial = 00)" is unclear.

We understand why this was unclear, and we have clarified this within the methods section in lines 674-680 (previously line 577):

> "Subsequently, smORFs are filtered to retain only those that meet the following criteria: a maximum length of 100 aa, presence of a ribosome binding site (RBS) motif, and completeness (i.e. presence of start and stop codon (partial=00)). Because smORFs are short and prone to spurious prediction, we applied the completeness check and RBS filter as a conservative strategy to select genuinely translated ORFs. Prodigal learns a genome-specific RBS, starting from a Shine-Dalgarno (SD) assumption and switching to a more general motif finder when SD usage is weak, so it captures both canonical and non-canonical RBS patterns[14]."

12. Line 365: Typographical error: missing space in "SEPsdetected"

Thank you for bringing this to our attention; this typo has been corrected".

Reviewer #1 (Remarks on code availability):

13. The documentation is minimal and not sufficient to reproduce the results.

We appreciate the reviewer for this comment, as we see now that we missed some crucial information from both the methods and the repository. We have included all the parameters that were missing from the methods as well as updated the repository to include more detail and make reproducibility more straightforward. In the section "smORF prediction pipeline" (several sections in lines 665-710) we described the parameters used for AntiFam, Infernal and Aragorn, as well as the MMseqs2 parameters for dereplication and for the taxonomy annotation. In addition, we also included the MMseqs2 parameters in the "Metaproteomics database construction section" (several sections in lines 712-751), which were also missing.

Please see **comment 2** for additional information.

Reviewer #2 (Remarks to the Author):

14. Davin et al. present a careful study of the metaproteome of the human gut microbiome. The research is very interesting and the manuscript is well written. The field certainly needs improved workflows and standardization of protocols, and I commend the authors on their work on these. Two aspects that are quite novel are firstly use of the Orbitrap Astral technology which seems to allow detection of many more proteins. Secondly, including statistics on abundance is very interesting and quite novel. Perhaps the novel aspects could be highlighted more clearly e.g. in the abstract, and perhaps the increase over previous studies could be noted.

Thank you for your thorough review and comments on our manuscript. We are happy to hear your appreciation for the manuscript and study. To address your comment about emphasizing novelty and comparing it to previous studies, we have reworded the abstract in lines 53-55:

> "Applied to human gut microbiomes, this approach resulted in the largest number of detected SEPs to date, allowing identification of over 25,000 SEPs in the metaproteome, alongside the measurements of the larger proteins. Our multi-omics integrative strategy is critical for advancing human metaproteome research. It also provides a generalizable strategy for comprehensive SEP discovery across diverse microbial ecosystems greatly expanding the previously hidden proteomic landscape."

And additionally, in the discussion we stress the novelty of our work by comparing our dataset to past research articles in lines 566-569:

> "Applied to human gut microbiome samples, this workflow coupled with proteomic validation produced, to our knowledge, the deepest measurements of SEPs, with careful FDR control while utilizing the high-performance Orbitrap Astral mass spectrometer, and outperformed current untargeted and size-exclusion methods[7,54,55]."

More specifically, this manuscript presents a workflow that accurately allows for microbial smORF prediction and SEP detection and has generated the largest known gut-microbiome SEP database to date. Detection of these SEPs is done without compromising the detection of the larger proteins per sample, and out competes existing research papers both using size-exclusion and untargeted methods listed below (and cited on line 569):

https://www.frontiersin.org/journals/genetics/articles/10.3389/fgene.2021.713400/

https://link.springer.com/article/10.1186/s40168-020-00981-z

https://www.cell.com/cell/fulltext/S0092-8674(19)30781-0

Substantive points

15. There is very little in the methods section about the underlying genomes (contigs) used to build the database, usually referred to as "metagenome assembled genomes" (MAGs) - please clarify the number of genomes or contigs present and their quality statistics. Similarly, there is almost nothing in the methods regarding taxonomic assignment. What does it mean to "map to a taxon" here? Is this based on the contigs, or on EGGNOG, or something else?

We appreciate your comment. The methods sections have now been revised to include more information on how the databases were built as well as clarification of the taxonomic assignment. We have included all the parameters that were missing from the methods, as well as updated the repository to include more details and make reproducibility more straightforward. In the section "smORF prediction pipeline" (in lines 659-704) we have described the parameters used for AntiFam, Infernal and Aragorn, as well as the MMseqs2 parameters for dereplication and for the taxonomy annotation, which now is more clearly explained. In addition, we also included the MMseqs2 parameters in the "Metaproteomics database construction section" (starting on line 705), which were also missing.

We are not using MAGs, we are working with contigs. We included the summary statistics in the methods in lines 659-660:

> "The metagenomic and metatranscriptomic per sample contig statistics can be found in **Supplemental Table 12**."

And the taxonomic assignment can be found in the methods in lines 698-700:

> "The predicted smORFs taxonomic annotations are obtained by using MMseqs2 (version 15.6f452; RRID:SCR_022962) with GTDB (version 10-RS226). The parameters used are easy-taxonomy --tax-lineage 1 -v 1 --report-mode 1."

The taxonomic assignments for the peptides were extrapolated from the search databases. The peptide taxonomic summed abundance is explained in the lines 785-787:

> “Taxa (extrapolated from the database) were quantified by summing the intensities of all peptides that uniquely mapped to each taxon. Protein, and taxonomy quantification are further explained in **Supplemental Method 6**.”

With further explanation in **Supplemental Method 6**:

> “Taxa (extrapolated from the database)) were quantified by summing the intensities of all peptides that uniquely map to each taxon (If a peptide was mapped to multiple proteins, the lowest (most-specific) shared taxonomic rank among those proteins was identified. The peptide’s abundance is then assigned to that common level and incorporated into the summed abundance for each of the corresponding taxonomic levels). A single peptides intensity was added to all levels in the taxonomic hierarchy that the peptide could be uniquely attributed to. Taxon intensities were then divided by the sum of all peptide intensities in a sample to get the fraction of observed intensity attributable to a taxon. Functions were quantified by summing the intensities of all peptides that coherently mapped to each function. A coherent relationship is one in which all proteins that could generate a peptide are annotated with the function in question. This does not require that the peptide uniquely maps to a function. A single peptide can have a coherent relationship with multiple functions, even those of the same type, and its intensity would be added to all of those functions. As with the taxon quantification function, sums were divided by the total observed intensity in each sample.”

16. A comparison to previous catalogues such as the GMSC (how much overlap is there?) would be relevant, if there is space for this.

Thank you to the reviewer for this comment; we agree that this information is relevant for the paper. Therefore, this information has been added to the manuscript in the results in lines 288-297:

> “To assess the coverage of our predicted smORFs in a global reference resource, we aligned all smORFs in our metaproteomics database against the Global Microbial smORF Catalog (GMSC) using GMSC-mapper[8,32]. Overall, 3,382,553 smORFs (89.95%) had homologs in GMSC, indicating substantial overlap with the current global catalogue; of these, 706,802 (20.90%) were classified as high quality, reflecting homology to a reference smORF that passed stringent confidence filters in the GMSC. Habitat, taxonomic, and

domain annotations were transferred from matched GMSC entries: most smORFs were associated with multiple habitats (88.79%), predominantly the human gut (98.66%), whereas 97.08% received taxonomic annotations, and 12.53% contained conserved domains. Notably, metaproteomics data provided experimental support for 1,585 predicted smORFs lacking homologs in GMSC."

In the methods in lines 746-748:

"Non-redundant smORFs from our database were queried against GMSC v1.0 using GMSC-mapper[8,32]. Habitat, taxonomy, quality, and CDD annotations transferred from hits. Full details in **Supplemental Method 3**."

and in the discussion in lines 502-509:

"Mapping our non-redundant predicted smORFs to the GMSC revealed that 89.95% have detectable homologs, underscoring the strong concordance between our predictions and existing global smORF resources. Importantly, the remaining smORFs absent from GMSC are unlikely to represent false positives, as they passed multiple stringent filtering steps, including Antifam, Aragorn, and Infernal screening, as well as checks for partiality and the presence of ribosome binding site motifs. Moreover, a subset of these GMSC-absent smORFs (1,585) was independently validated by our metaproteomics data, providing experimental support for the existence of potentially novel smORFs not yet captured in current reference catalogs."

17. This sentence "The detected taxonomic species account for less than 10% seen in the metagenomic data" is a key point which should be briefly explained. Is this due partly to the methods used for taxonomic assignment of smORFs, and/or perhaps low abundance of some species?

Your comment brings up some great considerations. This number is fairly typical for many publications that compare metagenome to metaproteome detections. The metagenomes are generally much deeper than the measured metaproteomes, due to several factors: 1) most importantly, the metagenomes measure total genomic inventory while the metaproteomes measure context-dependent proteome expression at the time of sampling, which can be greatly reduced relative to total genomic potential. 2) due to the lack of amplification methods, the dynamic range of the measured metaproteome is reduced relative to that achievable for the metagenome, and 3) high resolution taxonomic assignments are challenging in metaproteomics due to shared genomic inventories; thus, it is often difficult to attribute peptides/proteins to a specific species or strain relative to higher levels (genus, family, etc). This sentence has been re-worded for clarification in lines 534-541:

"The detected taxonomic species account for only a fraction of what is seen in the metagenomic data. Metagenomes are typically much deeper than the measure of metaproteomes because metagenomics provides total genomic inventories while

metaproteomes measure context-dependent protein expression, which is often greatly reduced relative to total genome potential. In addition, metaproteomics is unable to utilize amplification methods, limiting the dynamic range of the measurements, and furthermore, high-resolution taxonomic assignments are challenging in metaproteomics due to genomes within more-specific taxonomic ranks (i.e. family, or species) having high homology)."

18. The requirement for a ribosome binding site deserves brief discussion. What is the proportion of small proteins expected to have a RBS site?

We agree that a discussion about RBS sites is necessary. An RBS site was a requirement for our smORF prediction, so 100% of the predicted smORFs will have an RBS site. However, it has been previously reported that 73.8% of smORFs contain an RBS site (https://pmc.ncbi.nlm.nih.gov/articles/PMC11796252/), meaning that our filter excludes around 26.2% of potentially biologically relevant smORFs. While we acknowledge this conservative approach sacrifices some sensitivity, it was implemented as a critical quality control to minimize false positive predictions in our metaproteomics database. Including large numbers of unfiltered candidate smORFs substantially expands the search space during peptide-spectrum matching, which directly elevates false discovery rates (FDR). Our stringent RBS filtering prioritizes high-confidence predictions to maintain rigorous FDR control and minimize false positives that would otherwise compromise metaproteomic validation reliability. The filter's effectiveness is demonstrated by our pipeline's superior recovery of EcoCyc-validated smORFs compared to smORFinder (25.6% vs 9.8% recovery of ≤50 aa proteins at relaxed criteria; **Supplemental Table 1**), confirming that our quality filtering enhances true biological signal detection rather than obscuring it., At the suggestion of the reviewer, we have added more information about this in lines 496-499:

> "RBS motifs were used as a validating metric because it has been previously reported that 73.8% of smORFs contain an RBS site[39]. While this requirement could miss some potential smORFs, it was a necessary quality filter to minimize false positives."

Minor points

19. Fig 3D - I suggest (at your discretion) using a different visualisation to allow including category "S" - e.g. bar plots on log scale. It is unclear why unclassified orders needed to be excluded, they could be an additional row.

Thank you for pointing this out. This category has been included in a revised version of **Fig. 3D**.

20. It is stated that “Overall, the taxonomic make-up of these samples is relatively consistent with the global proteome taxonomic profiles, validating that we are not observing spurious small protein hits but rather biologically meaningful patterns” but it is not clear how this follows. Spurious proteins would also follow the taxonomic distribution of the underlying genomes - please clarify.

Thank you for requesting clarification on this sentence, as we understand it may be confusing. While small proteins hits to random genome matches could show a weak correlation, spurious small protein hits arising from random genome matches would be expected to show an incoherent correlation with the actually expressed proteome. The intention of this statement was not to compare the SEP profile to the metagenomic taxonomic composition but rather compare the SEP metaproteome to the global metaproteome derived from the same samples. This comparison shows that SEP detection follows the same organism-level abundance patterns as the broader proteome, supporting the biological relevance of these small proteins.

To avoid confusion, we have reworded this sentence to explicitly state that this is a comparison between SEP and global metaproteomics profiles rather than SEPs and the metagenomic profile as seen in lines 409-412:

> “Overall, the taxonomic composition of the small protein identifications is consistent with that observed in the global metaproteome (including proteins >100 aa), validating that we are not observing spurious small protein hits but rather biologically meaningful patterns (**Supplemental Table 11**).”

21. Line 333: “does not fit within” - typo.

Thank you for catching this. The typo has been corrected on line 391 (previously line 333).

<u>Reviewer #2 (Remarks on code availability):</u>

22. I have only briefly reviewed the code and note that for the bash script to run the snakemake workflow, the directories appear to be hard coded, they should probably be passed as arguments instead if it is to be usable by others.

The repository has been updated to address this concern. The config file, bash script, and README.md files have been updated to include more detailed information regarding the usage of the pipeline.

Reviewer Comments Response

We appreciate the reviewer's feedback. We have included text corrections below as well as a "revised" manuscript pdf with changes highlighted so that they may be easily identified.

Reviewer 1

1. It seems questionable to search for fully tryptic peptides given the small size of ORFs. One would expect many peptides to be semi-tryptic or not tryptic at all due to the very few opportunities for trypsin digestion.

We appreciate and agree with the reviewer's observation that small open reading frames (smORFs) may contain fewer tryptic cleavage sites. In fact, we did consider this aspect during experimental design but decided to intentionally restrict the database search to fully tryptic peptides to maintain stringent identification confidence.

While smaller proteins can indeed have fewer cleavage sites, the probability of generating tryptic peptides in short proteins is still relatively high. On average, protein sequences contain a lysine or arginine approximately every ten amino acids. Thus, for SEP ranging from 50-100 amino acids, this would suggest that each of these would have approximately 5-10 possible tryptic cleavage sites per SEP. However, in response to the reviewer's concern here, we did an additional evaluation of our search databases and found that 98.21% of small proteins (≥100 aa) have at least one tryptic peptide. Furthermore, 96.8% of small proteins that are 50 aa or smaller yield a tryptic peptide.

For this study, our goal was to take a conservative approach to identify smORF encoded proteins (SEPs) within the metaproteome. Allowing semi-tryptic or non-specific cleavage sites dramatically increases the theoretical search space. Importantly, we realize that in proteomics workflows, expanding enzyme specificity substantially increases the number of candidate peptide sequences considered during database searching, thereby reducing score discrimination and increasing the probability of false peptide spectral matches. While this challenge is somewhat less severe in single-organism proteomes, the impact is substantially greater in metaproteomic datasets, where database sizes are already large. Expanding enzymatic specificity in this context can increase the search space by an order of magnitude. For this reason, most large-scale proteomics workflows usually restrict searches only to fully tryptic peptides to maintain robust false discovery rate (FDR) control.

Therefore, because the large majority of SEPs in our database contain a tryptic peptide, and SEP discovery already requires searching expanded metaproteomic datasets, we felt that relaxing enzymatic specificity would further inflate the search space and compromise statistical confidence in peptide identifications without any significant gain (Fu, 2012; Aggarwal et al., 2022).

We fully acknowledge that, with our strategy, we are not identifying all SEPs but based on the metrics above, we feel that we are detecting the large majority, even with these somewhat conservative search criteria. In order to account for the reviewer's point, we have added new text in the methods section from lines 777 to 780 to better clarify this point:

"In the search databases, 98.21% of small proteins (≥100 aa) contained at least one tryptic peptide. Because of this, searches were kept to fully tryptic peptides, and not expanded for semi or non-tryptic peptides, to limit search database size, which in turn would greatly inflate false positive identifications."

2. The benchmark proposed is highly problematic. While the methods proposed may be more sensitive, a tool that calls everything an ORF would also appear more sensitive. The error rate (or detection rate) of predictions from smORFinder is vastly better: 16 out of 17 compared to the authors' tool (42 out of 450). The authors also do not provide any FDR assessment for the results from either tool. Protein identification numbers cannot be meaningfully compared without reporting FDRs. The authors should have at least provided performance curves (number of identifications) for both tools at different and comparable FDR thresholds.

   At this stage, I remain far from convinced that the proposed tool represents an improvement in validation compared to the other tool, contrary to the authors' claims. Furthermore, the benchmarking remains limited, as only a single tool is tested across two datasets.

We thank the reviewer for their comment and would like to clarify a few aspects of the benchmarking.

Both tools were evaluated against the same EcoCyc reference dataset. While our pipeline can predict smORFs up to 100 amino acids, we explicitly restricted comparisons to ≤50 amino acids when benchmarking against smORFinder. Therefore, the comparison is performed on a matched space, and the improved recovery is not due to an expanded prediction range. We also report results for smORFs of 50 – 100 amino acids to highlight a key biological finding of this study: a substantial fraction of validated small proteins falls

within the 50–100 amino acid range, which is not captured by smORFinder. This extended range is therefore biologically relevant rather than contributing to inflating performance.

Using the EcoCyc benchmark, smORFinder correctly predicted 17 smORFs ≤50 aa, whereas our pipeline predicted 91 smORFs within the same size range (450 total ≤100 aa). Using MMseqs2 validation against EcoCyc with 100% identity and coverage, smORFinder did not recover any validated smORFs (0/164), and only recovered 16/164 (9.8%) when allowing 90% coverage. In contrast, our pipeline recovered 41/164 (25.0%) validated smORFs ≤50 aa under stringent criteria (100% identity and 100% coverage), and 343/536 (63.8%) when considering smORFs up to 100 aa. In addition, we would like to clarify that the EcoCyc benchmark constitutes a gene-level evaluation against a curated set of experimentally validated smORFs and is therefore independent of metaproteomics-based false discovery rate considerations. In this context, reporting raw recovery numbers is appropriate, as the EcoCyc-reference set represents a ground truth.

On our co-assembled contigs, our prediction pipeline identified 524,743 smORFs, whereas smORFinder predicted 77,027 non-redundant smORFs ≤50 amino acids. When we next checked the overlap between the two methods using sequence-based matching (MMseqs2; 100% identity, ≥90% coverage), we found that 76,261 smORFs were predicted by both approaches. We next assessed metaproteomic support for these predictions. Among the shared smORFs, 628 were validated by metaproteomics. In contrast, our pipeline identified 1,912 metaproteomically supported smORFs in total, including 1,284 that were not predicted by smORFinder. These results indicate that, while our method recovers the vast majority of smORFinder predictions, it also identifies significantly more smORFs with experimental support within the same length constraints. Furthermore, our approach is not limited to ≤50 amino acids and extends to smORFs in the 50 – 100 amino acid range, which themselves are also supported by metaproteomic data (Figure 4D).

To clarify our evaluation procedure on the co-assembled contigs, both smORFinder and our pipeline were applied to the same input dataset to generate independent sets of predicted smORFs. We then quantified the overlap between the two methods using sequence-based matching (MMseqs2; 100% identity, ≥90% coverage), showing that 76,261 of 77,027 smORFinder predictions are also recovered by our pipeline. Metaproteomics was subsequently used as an orthogonal validation step to assess how many predicted smORFs from each method were supported by peptide evidence. As, to our knowledge, no metagenomic dataset with a fully metaproteomics-validated smORF ground truth is currently available, this approach provides a practical means to evaluate method performance on real data and to determine whether expanding the prediction space recovers additional experimentally supported smORFs.

We acknowledge that only one alternative tool was included in this comparison. However, smORFinder is currently, to our knowledge, the only tool available that is specifically designed for microbial smORF prediction in metagenomic data.

For clarification purposes, we have rewritten the main text using text which had already been described in the Supplementary Note 1 on lines 137-153:

"We benchmarked our pipeline against smORFinder using *E. coli* K-12 substrain MG1655 (536 EcoCyc-validated small proteins ≤100 aa, 164 ≤50 aa). SmORFinder predicted 17 smORFs ≤50 aa, while our prediction pipeline predicted 450 ≤100 aa of which 91 were ≤50 aa (**Supplemental Table 1**). Validation with MMseqs2 against EcoCyc, using 100% identity and coverage, found no matches among the smORFs predicted by smORFinder (0/164). When the coverage threshold was lowered to 90%, 16 of 164 smORFs (9.75%) were detected. In contrast, our pipeline identified more smORFs in EcoCyc at 100% identity and coverage: 41 of 164 (25%) for sequences ≤50 amino acids, and 343 of 536 (63.8%) for sequences ≤100 amino acids (**Supplemental Table 1**). On the co-assembled contigs, smORFinder predicted 77,027 non-redundant smORFs ≤50 aa, with 628 having metaproteomic identifications. Our prediction pipeline predicted 524,743 smORFs, with 76,261 also being predicted by smORFinder (MMseqs2 100% identity ≥90% coverage), and 1,912 having been identified by metaproteomics. The full results and parameters for this section can be found in **Supplemental Note 1**. Overall, our approach substantially improved small protein recovery and validation rates, yielding substantially higher sensitivity against EcoCyc-validated proteins and identifying a greater number of metaproteomics-supported smORFs when compared to smORFinder."

3. The FDRs reported in the study remain high and are in fact even higher now that the entrapment strategy is used and seem to be more representative of the data. The authors claim that a 5% FDR threshold is widely accepted and cite a single study from Proteomics. This claim is greatly exaggerated. This threshold is not widely accepted. While users can choose the FDR threshold they are willing to accept in their experiments, given the concerns regarding specificity, the authors should report all of their results at a 1% FDR.

We acknowledge the reviewer's concern regarding false discovery rate (FDR) thresholds and thus would like to clarify how FDR control was implemented in our study. All database search results were filtered at a strict 1% peptide-spectrum match (PSM) and 1% peptide-level FDR using a target-decoy strategy at the global dataset level, including the smORF-derived peptides.

In addition to this global filtering, we calculated SEP-specific FDR values using an entrapment strategy. These values were not used as filtering thresholds but instead

reported as metrics to assess how false identifications are distributed within this particular subset of the search space. When large databases are searched, false positives often distribute unevenly across proteome subsets, meaning that the effective FDR for a subset may appear higher than the global FDR, even when the entire dataset is controlled at 1%. This phenomenon has been previously described and reflects a known consequence of expanding proteomic search spaces and has long been something of importance to take into consideration (Fu, 2012).

In fact, this behavior is not unique to expanding a dataset for SEP detection but can be observed broadly in proteomics when analyzing a variety of subsets of the proteome.

The intent of reporting the smORF-specific FDR values is to transparently illustrate this phenomenon (which is a more thorough approach than what is commonly reported in the literature), and to emphasize this phenomenon when expanding search spaces in any manner, including but not exclusive to small protein discovery. The SEP-specific estimates simply provide additional context for interpreting small protein identifications and do not alter the primary filtering criteria used in our study.

We agree that 1% FDR represents a stringent and commonly accepted threshold standard, which is why this threshold applied throughout the searches in this paper. We also recognise that there are still numerous papers that use a 5% FDR as their confidence threshold, so our ~5% FDR for the SEPs from the entrapment database is not unusually high, even for this subset of the global database. To support this claim and address the reviewer's concern, we have expanded our citations of papers that utilize a 5% FDR:

- Lazear et al., 2023
- Li et al., 2022
- Ishino et al., 2026
- Pandey et al., 2023
- Menon et al., 2024

In total, the objective of this study was to identify small, encoded proteins (SEPs) alongside the broader global proteome, allowing them to be evaluated within the same experimental framework as larger proteins. To address the reviewer's concerns, we have clarified this limitation and recommendation in the revised manuscript on lines 782 to 785:

"Though the global searches, including the SEP identifications, were controlled at a 1% peptide level FDR, subsets of proteomes can carry differing FDRs. In order to interrogate the composition of the SEP detection, a SEP-specific false discovery rate (FDR) at the peptide level was calculated and evaluated with an entrapment database."

And on lines 793 to 797:

> "The intent of reporting the smORF-specific FDR values is to transparently illustrate and emphasise this phenomenon when expanding search spaces in any manner, including but not exclusive to small protein discovery. The SEP-specific estimates simply provide additional context for interpreting small protein identifications and do not alter the primary filtering criteria used in the study."

4. (Remarks on code availability): The authors have improved the documentation.

We appreciate the acknowledgement of appropriate revision.

Reviewer 2

5. My previous review comments have been addressed well - well done to the authors on the careful responses to reviewer comments, and the detailed work in this study.

We appreciate the acknowledgement of appropriate revision.

6. The sentence "high-resolution taxonomic assignments are challenging in metaproteomics due to genomes within more-specific taxonomic ranks (i.e. family, or species) having high homology)" should be corrected to something like "high similarity" as homology, technically, does not differ by degrees.

We agree with your statement, and this has been changed in the sentence from line 541-544:

"In addition, metaproteomics is unable to utilize amplification methods, limiting the dynamic range of the measurements, and furthermore, high-resolution taxonomic assignments are challenging in metaproteomics due to genomes within more-specific taxonomic ranks (i.e. family, or species) having high similarity."

7. Line 213: typo – coverages

Thank you for catching this typo. It has been corrected on in the sentence from line 214 to 216:

"The latter thresholds were more relaxed than the parameters typically used in microbiome smORF studies, which commonly use identities and coverages between 90% and 95%."

Citations

Aggarwal, S., Raj, A., Kumar, D., Dash, D., & Yadav, A. K. (2022). False discovery rate: the Achilles' heel of proteogenomics. *Brief Bioinform*, *23*(5). https://doi.org/10.1093/bib/bbac163

Fu, Y. (2012). Bayesian false discovery rates for post-translational modification proteomics. *Statistics and Its Interface*, *5*(1), 47–59.

Ishino, T., Watanabe, T., Tokita, S., Ueda, Y., Kawase, K., Takano, Y., Thu, Y. M., Suzuki, Y., Owa, C., Inozume, T., Zhou, W., Nagasaki, J., Kochin, V., Ueno, T., Kojima, S., Honobe-Tabuchi, A., Kawamura, T., Ohnuma, T., Matsuzawa, T.,…Togashi, Y. (2026). Immunopeptidomics combined with full-length transcriptomics uncovers diverse neoantigens. *Cell Reports*, *45*(1), 116781. https://doi.org/https://doi.org/10.1016/j.celrep.2025.116781

Lazear, M. R., Remsberg, J. R., Jaeger, M. G., Rothamel, K., Her, H. L., DeMeester, K. E., Njomen, E., Hogg, S. J., Rahman, J., Whitby, L. R., Won, S. J., Schafroth, M. A., Ogasawara, D., Yokoyama, M., Lindsey, G. L., Li, H., Germain, J., Barbas, S., Vaughan, J.,…Cravatt, B. F. (2023). Proteomic discovery of chemical probes that perturb protein complexes in human cells. *Mol Cell*, *83*(10), 1725–1742.e1712. https://doi.org/10.1016/j.molcel.2023.03.026

Li, L., Niu, M., Erickson, A., Luo, J., Rowbotham, K., Guo, K., Huang, H., Li, Y., Jiang, Y., Hur, J., Liu, C., Peng, J., & Wang, X. (2022). SMAP is a pipeline for sample matching in proteogenomics. *Nature Communications*, *13*(1), 744. https://doi.org/10.1038/s41467-022-28411-8

Menon, T., Illing, P. T., Chaurasia, P., McQuilten, H. A., Shepherd, C., Rowntree, L. C., Petersen, J., Littler, D. R., Khuu, G., Huang, Z., Allen, L. F., Rockman, S., Crowe, J., Flanagan, K. L., Wakim, L. M., Nguyen, T. H. O., Mifsud, N. A., Rossjohn, J., Purcell, A. W.,…Kedzierska, K. (2024). CD8+ T-cell responses towards conserved influenza B virus epitopes across anatomical sites and age. *Nature Communications*, *15*(1), 3387. https://doi.org/10.1038/s41467-024-47576-y

Pandey, K., Wang, S. S., Mifsud, N. A., Faridi, P., Davenport, A. J., Webb, A. I., Sandow, J. J., Ayala, R., Monje, M., Cross, R. S., Ramarathinam, S. H., Jenkins, M. R., & Purcell, A. W. (2023). A combined immunopeptidomics, proteomics, and cell surface proteomics approach to identify immunotherapy targets for diffuse intrinsic pontine glioma [Original Research]. *Frontiers in Oncology*, *Volume 13 - 2023*. https://doi.org/10.3389/fonc.2023.1192448
